# Flap endonuclease 1 repairs DNA-protein cross-links via ADP-ribosylation–dependent mechanisms

**DOI:** 10.1126/sciadv.ads2919

**Published:** 2025-01-10

**Authors:** Yilun Sun, Lisa M. Jenkins, Lara H. El Touny, Linying Zhu, Xi Yang, Ukhyun Jo, Lauren Escobedo, Tapan K. Maity, Liton Kumar Saha, Isabel Uribe, Sourav Saha, Shunichi Takeda, Anthony K. L. Leung, Ken Cheng, Yves Pommier

**Affiliations:** ^1^ Department of Pharmacology, Physiology and Drug Development, University of Maryland School of Medicine, Baltimore, MD 21201, USA.; ^2^ University of Maryland Marlene and Stewart Greenebaum NCI Comprehensive Cancer Center, Baltimore, MD 21201, USA.; ^3^ Developmental Therapeutics Branch, Center for Cancer Research, National Cancer Institute, National Institute of Health, Bethesda, MD 20892, USA.; ^4^ Mass Spectrometry Section, Collaborative Protein Technology Resource, National Cancer Institute, National Institute of Health, Bethesda, MD 20892, USA.; ^5^ Function Genomics Laboratory, National Center for Advancing Translational Sciences, National Institute of Health, Rockville, MD 20850, USA.; ^6^ Department of Biochemistry and Molecular Biology, Bloomberg School of Public Health, Johns Hopkins University, Baltimore, MD 21205, USA.; ^7^ Shenzhen University School of Medicine, Shenzhen, Guangdong, China.

## Abstract

DNA-protein cross-links (DPCs) are among the most detrimental genomic lesions. They are ubiquitously produced by formaldehyde (FA), and failure to repair FA-induced DPCs blocks chromatin-based processes, leading to neurodegeneration and cancer. The type, structure, and repair of FA-induced DPCs remain largely unknown. Here, we profiled the proteome of FA-induced DPCs and found that flap endonuclease 1 (FEN1) resolves FA-induced DPCs. We revealed that FA also damages DNA bases adjoining the DPCs, leading to DPC-conjugated 5′ flap structures via the base excision repair (BER) pathway. We also found that FEN1 repairs enzymatic topoisomerase II (TOP2)–DPCs. Furthermore, we report that both FA-induced and TOP2-DPCs are adenosine diphosphate (ADP) ribosylated by poly(ADP-ribose) polymerase 1 (PARP1). PARylation of the DPCs in association with FEN1 PARylation at residue E285 is required for the recruitment of FEN1. Our work unveils the identity of proteins forming FA-induced DPCs and a previously unrecognized PARP1-FEN1 nuclease pathway repairing both FA- and TOP2-DPCs.

## Teaser

FEN1 removes 5′ overhanging DNA flaps cross-linked with denatured or proteolyzed proteins via PARP1-mediated ADP-ribosylation.

## INTRODUCTION

DNA-protein interactions are at the heart of cellular functions (*1*). In eukaryotic cells, genomic DNA is wrapped around histone octamers to allow chromosomal packaging. In addition, binding of a wide range of enzymes to DNA directs replication, transcription, and chromosome remodeling and governs DNA damage responses. Because of their fundamental significance in all cellular processes involving DNA, disruptions of dynamic DNA-protein interactions have detrimental biological consequences (*2*).

Chromatin-interacting proteins are prone to covalent trapping on DNA upon exposure to endogenous DNA lesions or exogenous (environmental and chemotherapeutic) agents (*3*–*6*), leading to the formation of DNA-protein cross-links (DPCs). According to their chemical nature, DPCs can be divided into two classes. First, DNA-protein covalent conjugates that are normal enzymatic catalytic intermediates of multiple enzymes (e.g., topoisomerases, tyrosyl-DNA–phosphodiesterase I, and DNA and RNA polymerases) can be trapped under ubiquitous circumstances including enzyme malfunctions, exposure to endogenous DNA damage, and treatments with their respective inhibitors (*7*, *8*). Second, nonenzymatic DPCs are caused by the cross-linking of proteins in the vicinity of DNA by endogenous reactive metabolites as well as exogenous agents. As a result of endogenous exposure to reactive oxygen species, lipid peroxidation products, as well as normal cellular metabolism, in the absence of efficient DNA repair, nonenzymatic DPCs progressively accumulate in the brain and many other tissues (*9*). Because of their considerable size and their helix-distorting nature, DPCs interfere with the progression of replication and transcription machineries and hence hamper the faithful expression of genetic information. DPC-generated DNA damage induces cancer, neurodegeneration, immunodeficiency, hepatoxicity, and premature aging (*5*, *10*–*12*).

A prominent source of endogenous nonenzymatic DPCs is aldehydes, which are highly reactive and cross-link proteins adjacent to DNA. Formaldehyde (FA) is generated as by-product of multiple metabolic processes including methanol metabolism, histone demethylation, and lipid peroxidation; FA is also an important environmental pollutant (*5*). It is found at high concentrations in human plasma and tissues, implying that DPCs are continuously induced by FA and cells must constantly overcome DPC-induced damage.

The redundant mechanisms and multiple repair proteins that remove DPCs are increasingly being elucidated, especially for the enzymatic DPCs generated by DNA topoisomerases (*4*, *6*, *9*). While the complete array of DPC repair pathways remains to be fully unraveled, recent evidence has established that debulking of DPCs can be achieved by diverse proteases depending on the cellular context. The DNA-dependent metalloprotease SPRTN (Wss1 in yeast) acts on enzymatic and nonenzymatic DPCs during replication as a component of the replisome (*13*–*15*), while the 26*S* proteasome digests DPCs both during and outside of the S phase (*16*–*20*). In addition to SPRTN, several proteases, such as ACRC (*21*), also known as germ cell nuclear antigen (*22*), FAM111A and FAM111B, (*23*, *24*), and DDI1 and DDI2 (*25*, *26*) have recently been found and linked to DPC proteolysis.

In the case of FA-induced DPCs, the methylene bonds formed by FA between the amino groups of DNA bases and the lysine ϵ-amino groups of proteins (secondary amine) are not substrates for peptidases. Thus, cells must use a nucleolytic process to remove the DPCs. To find such repair nucleases, we first developed a mass spectrometry (MS)–based method to identify the proteins that form DPCs upon exposure to FA. This approach led to the identification of DNA processing enzymes {TOP1, TOP2α, TOP2β, DNMT1, and poly[adenosine diphosphate (ADP)–ribose] polymerase1 (PARP1)} as some of the most abundant DPC-associated proteins generated by FA. Next, we performed an unbiased RNA interference (RNAi) screen and our modified RADAR (rapid approach to DNA adduct recovery) assay (*17*) and identified flap endonuclease 1 (FEN1) as critical for the resolution of FA-induced DPCs. Flaps are single-stranded DNA structures arising during replication on the lagging strand, long-patch base excision repair (BER) or when the replication machinery encounters damaged DNA. FEN1 cleaves 5′-flap structures to generate nicks and initiate DNA repair (*27*). As FEN1 is a replication-associated nuclease also involved in Okazaki fragment maturation as well as a prominent BER enzyme (*28*, *29*), we hypothesized and herein demonstrate that FEN1 excises DPCs formed within a DNA flap generated by the BER pathway or within the RNA-DNA hybrid flaps of Okazaki fragments.

In addition, we tested whether that FEN1 also repairs the cellular enzymatic topoisomerase II (TOP2)–DPCs as the TOP2 homodimer breaks both DNA strands and forms a covalent enzyme-DNA intermediate termed TOP2 cleavage complex (*30*). We found that FEN1 provides a salvage route to excise both the TOP2α- and β- (two isozymes in mammals)–DPCs independently of the previously known SPRTN and TDP2 pathway (*19*, *20*).

Considering that PARylation plays a central role in sensing DNA damage (*31*) and that we found that PARP1-DPCs were among the most abundant DPCs induced by FA, we conjectured that PARylation participates in DPC repair. Accordingly, by blocking PARG (PAR glycohydrolase) (*32*), we demonstrate the transient PARylation of both the FA-induced DPCs and the etoposide (ETOP)–induced TOP2-DPCs. We also show that FEN1 is PARylated upon FA exposure at its E285 amino acid residue and that this PARylation is crucial for recruiting FEN1 to DPCs.

## RESULTS

### Coupling in ICE bioassay and quantitative MS identified the FA-induced DPC proteome

Pathway choice for DNA damage repair is largely contingent on the cellular context (replication, transcription, chromatin remodeling, chromosome segregation, etc.) (*33*). Given the heterogeneity of the nonenzymatic DPCs induced by FA, the repair of those DPCs remains only partially known. Elucidating the identity of the proteins involved in the DPCs is therefore important (*34*, *35*).

**Fig. 1.**
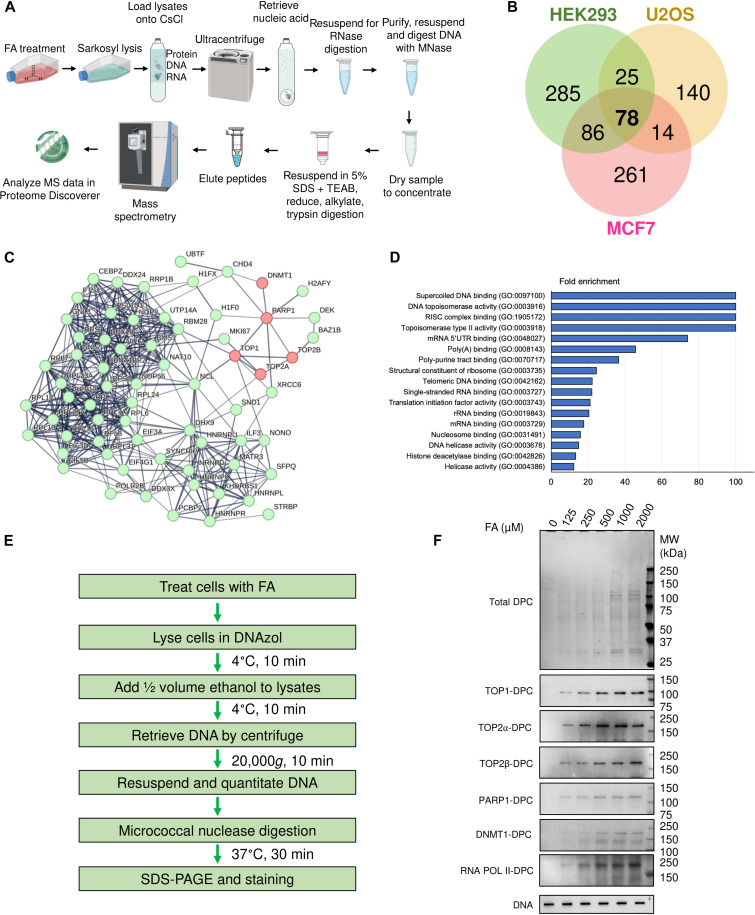
The ICE-MS method identified the FA-induced DPC proteome. (**A**) Experimental scheme of ICE-MS profiling of DPCs induced by FA. This figure was created with BioRender.com. (**B**) Venn diagrams depicting the number of DNA–cross-linked proteins up-regulated by FA (a 10-fold increase over control) across the indicated three cell lines. (**C**) Common DNA–cross-linked proteins generated by FA across the three cell lines are shown as network analysis using the STRING database (interaction confidence setting of 0.7). Proteins not connected to the network were omitted. Proteins that form enzymatic DPCs are highlighted in light red. (**D**) The common FA-induced protein adducts across the three cell lines were mapped to the human proteome, which was annotated with Gene Ontology (GO) molecular function. Enrichment analysis was performed by fold change measurement. (**E**) Experimental scheme of the modified RADAR assay for DPC detection. (**F**) The modified RADAR assay was performed in human embryonic kidney (HEK) 293 cells treated with FA at the indicated concentrations for 2 hours. Ten micrograms of samples was digested with 100 U of micrococcal nuclease and subjected to SDS-PAGE electrophoresis. Total DPCs were detected with Coomassie stain, and individual DPCs were probed with antibodies targeting the corresponding proteins. Two micrograms of samples without micrococcal nuclease digestion was subjected to slot-blot and probed with anti-DNA antibody as loading control.

Because a major challenge to the profiling nonenzymatic DPCs is their purification, we developed an approach by isolating FA-induced DPCs using cesium chloride ultracentrifugation, a method derived from the in vivo complex of enzyme (ICE) assay (*36*). We used 500 μM FA to induce detectable DPCs because such a concentration is physiologically relevant according to the European Food Safety Authority (*37*) and the standard concentration that has been used for the DPC study (*13*, *14*, *21*). Following the release of the cross-linked proteins using micrococcal nuclease (*17*), we profiled the liberated proteins by MS analysis (Fig. 1A). On the basis of this ICE-MS method, we unveiled the proteome of FA-induced DPCs in three human cell lines [U2OS, human embryonic kidney (HEK) 293, and MCF7] and found that the most abundant proteins that form the DPCs are PARP1, TOP1 and TOP2, DNA methyltransferases including DNMT1, DNA and RNA polymerases, histones, and ribosomal proteins (Fig. 1, B to D; fig. S1A; and dataset S1), of which TOP1, TOP2, and DNMT1 as well as PARP1 are also known to form enzymatic DPCs after exposure to endogenous DNA lesions and their inhibitors. Proteins such as DNA replication licensing factor MCM3 and transcriptional repressor CTCF were cross-linked to DNA only in U2OS cells, whereas proteins such as DNA replication licensing factor MCM4 and transcription intermediary factor TRIM28 were cross-linked to DNA only in MCF7 cells and proteins such as DNA repair factor XPC were cross-linked to DNA only in HEK293 cells, suggesting the difference in replication, transcription, and DNA repair programs among cell lines (dataset S1).

Proteomic analyses of chromatin fractions in U2OS cells showed that exposure to FA did not markedly elevate chromatin enrichment of PARP1, TOP1 and TOP2, methyltransferases including DNMT1, DNA and RNA polymerases (dataset S2 and fig. S1B), indicating that FA specifically cross-links preexisting chromatin-bound proteins to DNA.

We confirmed our DPC findings by conducting a modified RADAR assay (*34*, *35*, *38*) to immunodetect the proteins forming cellular DPCs. Consistent with our MS results, we detected TOP1-, TOP2α- and β-, DNMT1-, PARP1-, as well as RNA Pol II–DPCs induced by FA in a dose-dependent manner (Fig. 1, E and F).

### FEN1 is a nucleolytic mechanism for nonenzymatic DPC repair

**Fig. 2.**
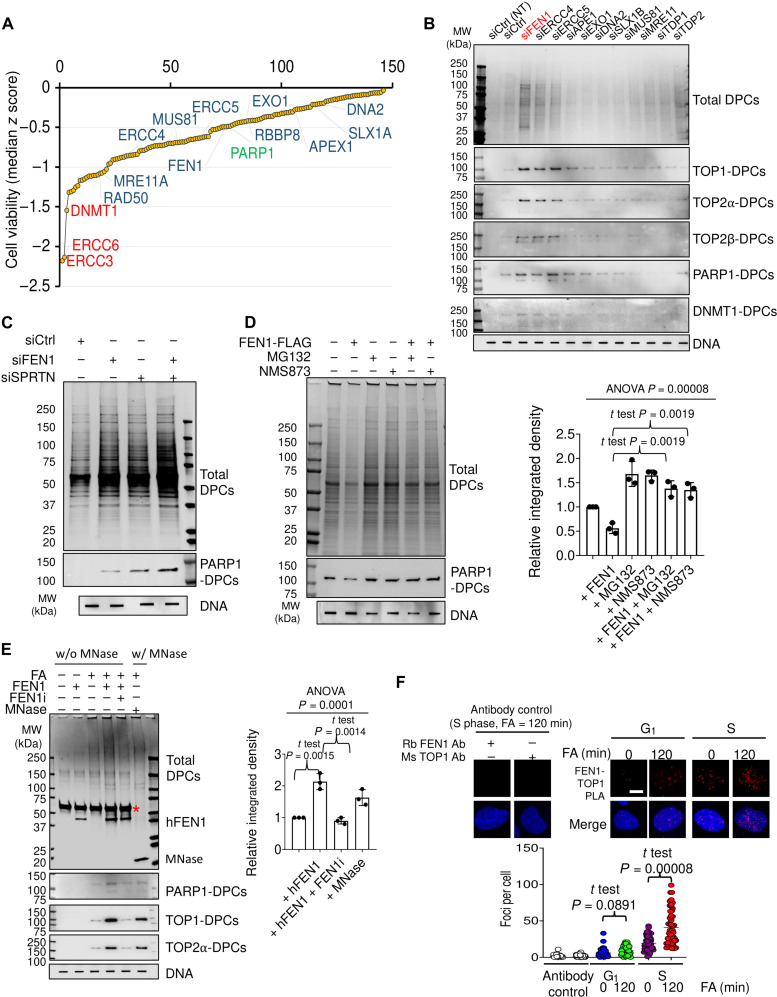
FEN1 is a major nucleolytic mechanism for nonenzymatic DPC repair. (**A**) Ranked distribution plots of median *z* scores obtained from RNAi screen in FA-treated/untreated MCF7 cells. (**B**) The modified RADAR assay in HEK293 cells transfected with the indicated siRNAs and treated with 400 μM FA for 4 hours. Total DPCs were detected with Coomassie stain. Two-micrograms of samples without micrococcal nuclease were subjected to slot-blot and probed with anti-DNA antibody as loading control. (**C**) The modified RADAR assay in HEK293 cells transfected with indicated siRNAs for 48 hours, followed by treatment with 400 μM FA for 2 hours. (**D**) Left, the modified RADAR assay in HT29 CRISPR KO cells treated with 10 μM MG132 or NMS873 for 1 hour and then cotreated with 400 μM FA for 2 hours. Right, densitometric analysis comparing total DPC signals generated from the modified RADAR assay in the left panel. *n* = 3 independent experiments. Data are presented as means ± SD. (**E**) Left, the modified RADAR assay in HEK293 cells treated with or without FA (400 μM, 2 hours). Samples were incubated with 500 nM recombinant human FEN1 or with 100 U of micrococcal at 37°C for 30 min. *, Bovine serum albumin (BSA), stabilizer in FEN1 cleavage buffer. Right, densitometric analysis comparing total DPC signals generated from the modified RADAR assays including blot shown in the left panel. *n* = 3 independent experiments. Data are presented as means ± SD. (**F**) Top, U2OS cells were arrested in the G_1_ phase by the double-thymidine block or released in the S phase 2 hours after the removal of thymidine. The cells were collected before or after FA treatment (400 μM, 120 min) for the PLA assay. Scale bar, 10 μm. Bottom, quantitation of PLA foci using Thunderstorm, a plugin of ImageJ. *n* = 200 biologically independent cells.

To identify the nuclease (s) required for FA-induced DPC repair, we conducted an RNAi high-throughput screen using a human ON-TARGETplus small-interfering RNA (siRNA) library directed at 240 genes involved in the DNA damage and repair pathways including nearly all the DNA repair nucleases. Following transfection of a set of four individual siRNAs for each gene in MCF7 cells in a 384-well plate format, we exposed the cells to 80 μM FA for 72 hours and subsequently measured their survival using the CellTiter-Glo luminescent assay. FA responses were converted to *z* scores from the four individual siRNA-transfected wells. The normalized *z* score data were plotted for each gene, and negative median *z* scores were considered synthetic-lethal interactions with FA treatment. ERCC3, ERCC6, and DNMT1 were identified as the highest hits. Down-regulation of genes encoding MRE11, ERCC4, MUS81, FEN1, ERCC5, EXO1, APEX1, SLX1A, and DNA2 nucleases were also found to confer hypersensitivity to FA (Fig. 2A and dataset S3).

To determine which nuclease helps cells survive by facilitating the removal of FA-generated DPCs, we used the modified RADAR assay and measured DPCs in cells transfected with siRNAs targeting the nucleases identified from the RNAi screen. Notably, down-regulation of FEN1 markedly increased the levels of DPCs as detected by Coomassie blue and immunoblotting against individual DPCs (Fig. 2B and fig. S2A). We next carried out epistasis analysis for FEN1 and SPRTN, a well-established DPC-targeted protease. Double knockdown (DKD) of FEN1 and SPRTN with their respective siRNAs showed an additional increase in the levels of FA-induced DPCs and in the sensitivity to FA in comparison with their respective single knockdown (Fig. 2C and fig. S2, B and C), suggesting FEN1 and SPRTN as redundant repair mechanisms for nonenzymatic DPC.

Under unperturbed conditions, CRISPR-Cas9 knocking out FEN1 in the HT29 colon cancer cell line led to an increase in the levels of DPCs in comparison with the isogenic WT cells (1.205 ± 0.115, *P* = 0.017; fig. S2, D and E), indicating a role of FEN1 in the removal of endogenous and spontaneous DPCs. In addition, SPRTN knocking down in the FEN1 KO cells led to a synergistic effect on the accumulation of endogenous DPCs (fig. S2F). However, FEN1 rescue expression in FEN1 KO cells failed to remove FA-induced DPCs in the presence of proteasome inhibitor or p97/VCP inhibitor (Fig. 2D), suggesting that proteolysis and/or denaturation of the protein component of the DPCs are required for FEN1 to act effectively toward the DPCs presumably by enabling FEN1 to traverse the otherwise bulky protein obstacle while tracking the 5′-end of the flap to the site of cleavage.

Next, we incubated DNA samples isolated by the RADAR assay with recombinant human FEN1 instead of micrococcal nuclease and subjected the samples to SDS–polyacrylamide gel electrophoresis (SDS-PAGE). We found that electrophoresis separated recombinant FEN1-treated RADAR samples but not the samples without FEN1 treatment (Fig. 2E), as proteins cross-linked to megabase DNA molecules are trapped in the wells of the SDS–polyacrylamide gels. These results suggested that FEN1 can act on denatured full-length DPCs that presumably result from p97/VCP processing (*39*). Blocking FEN1 endonuclease activity with its small-molecule inhibitor FEN1-IN-4 (FEN1i) (*40*) blocked the SDS-PAGE resolution (i.e., the DPC protein release) from the RADAR samples (Fig. 2E). Together, these results show that FEN1 processes DPCs using its endonuclease activity.

We next examined whether FEN1 and DPCs colocalize upon FA exposure. Using proximity ligation assay (PLA), we found that FEN1 and TOP1 interaction upon treatment with FA were markedly increased in U2OS cells released in S phase but not in G_1_ phase (Fig. 2F). Further, we monitored TOP1 subnuclear distribution with TOP1-HaloTag expression U2OS cells. In the S phase but not the G_1_ phase, TOP1 proteins were rapidly delocalized from nucleoli to the nucleoplasm and colocalized with FEN1 molecules following exposure to FA (fig. S2G), which is consistent with prior findings that TOP1-DPCs induced by TOP1 inhibitors are expelled from nucleoli and localized to nucleoplasm (*41*, *42*). Together, these data suggest that FEN1 repairs FA-induced DPCs in a replication-dependent manner.

### FEN1 cleaves DPC-harboring DNA flap substrates originated by oxidative DNA lesions

**Fig. 3.**
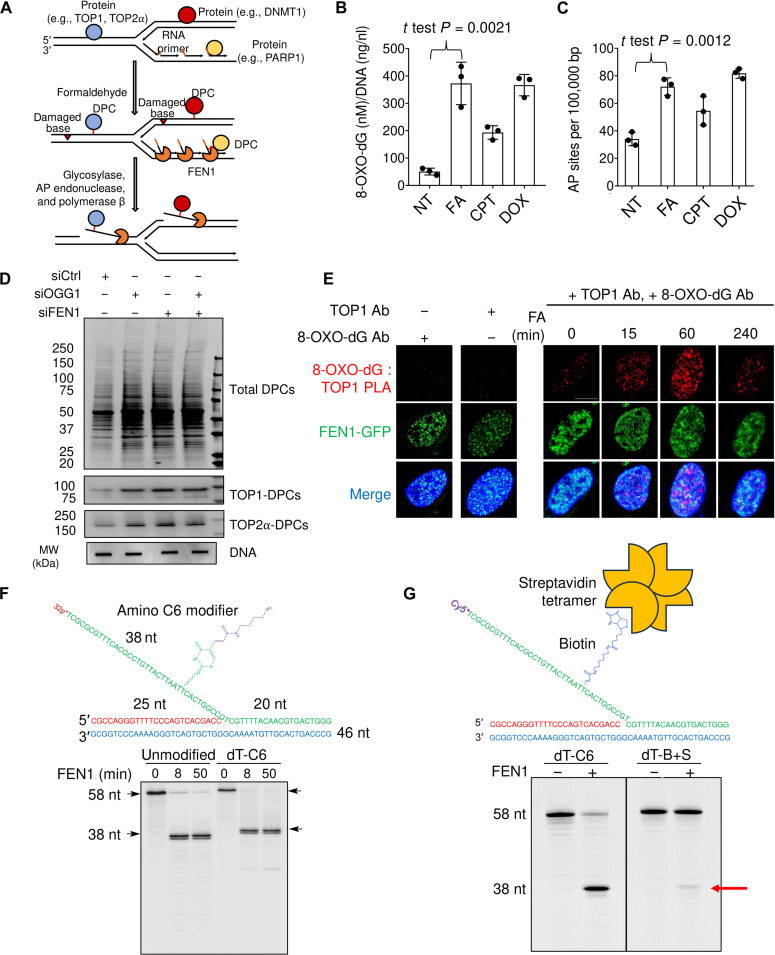
FEN1 cleaves DPC-harboring DNA flaps originated oxidative lesions. (**A**) Hypothetical model depicting the roles of FEN1 for the repair of FA-induced DPCs in replicating DNA. (**B**) DNA samples were isolated by RADAR assay in HEK293 cells treated with indicated agents (FA, 400 μM; TOP1 inhibitor camptothecin/CPT, 10 μM; TOP2 inhibitor doxorubicin/DOX, 10 μM) for 1 hour. Detection and quantitation of 8-OXO-dG in the samples were performed using HT 8-oxo-dG enzyme-linked immunosorbent assay kit. Error bars, SD. (**C**) The same DNA samples as in (B) were detected for apurinic or apyrimidinic (AP or abasic) sites using the AP Sites Quantitation Kit. (**D**) The modified RADAR assay in HEK293 cells transfected with the indicated siRNAs and subsequently treated with 400 μM FA for 2 hours. (**E**) The PLA assay in FEN1-GFP (green fluorescent protein) expression plasmid transfected U2OS cells, following 400 μM FA treatment for indicated periods to measure TOP1 and 8-OXO-dG using their respective antibodies. Scale bar, 10 μm. (**F**) Top, sequence and structure of double-stranded DNA substrate carrying a C6 amino modifier conjugated-5′-flap. *, ^32^P label. Bottom, activity assay testing recombinant human FEN1 toward unmodified and C6-modified DNA substrate shown in the top panel for indicated periods. ^32^P labeled DNA products following the activity assay were visualized by PAGE electrophoresis. (**G**) Top, sequence and schematic structure of the double-stranded DNA substrate carrying a biotin conjugated-5′-flap. The biotinylated substrate was incubated with recombinant streptavidin protein in a 4:1 ratio *, Cy5 dye. Bottom, activity assay testing recombinant human FEN1 toward the streptavidin-biotin–modified DNA substrate shown in the top panel for 30 min. Cy5-labeled DNA products following the activity assay were visualized by PAGE electrophoresis.

As FEN1 processes 5′-flap structures and FA generates DPCs without broken ends except when DPCs form close to the 5′-ends of Okazaki fragments in the lagging strand of newly synthesized DNA (*28*), we speculated that the no-break DPCs in the front of replication forks and on the leading strand behind the forks must be accompanied with 5′-DPC flaps in order for FEN1 to process these lesions (Fig. 3A). To test this hypothesis, we first examined whether FA induces base damage, as oxidative lesions can be converted to 5′-flaps by the BER pathway (*43*). Accordingly, we observed that FA elevated cellular levels of 8-oxo-deoxyguanine (8-OXO-dG) (Fig. 3B), one of the major products of DNA oxidation, and cellular levels of AP sites (apurinic/apyrimidinic site) (Fig. 3C), which are formed by the processing of damaged bases by DNA glycosylase activity as the first step of the BER pathway.

To further assess the role of the BER pathway in nonenzymatic DPC repair, we down-regulated OGG1 (8-oxoguanine DNA glycosylase) and performed modified RADAR and cell survival assays. Cells with OGG1 deficiency accumulated DPCs, whereas OGG1 and FEN1 DKD cells did not show further DPC accumulation (Fig. 3D and fig. S3, A and B) and were not sensitized to FA (fig. S3C), suggesting that OGG1 likely acts upstream of FEN1 during nonenzymatic DPC repair. Akin to knocking down OGG1, inhibition of Polβ activity using its inhibitor prunasin elevated the levels of FA-induced DPCs (fig. S3D). Next, we conducted PLA and observed colocalization between TOP1 and 8-OXO-dG upon FA treatment (Fig. 3E and fig. S3E). Because TOP1 acts in front of replication forks to relax accumulated positive supercoils (*7*), this suggests that FA generates adjoining base damage and DPCs ahead of replication forks, which is likely transformed into flaps harboring the DPCs by the BER pathway, eventually leading to their resolution by FEN1.

To directly test whether FEN1 can process DNA substrates with 5′-flap–containing DPCs, we designed a DNA substrate with a single-stranded flap internally conjugated with the amino modifier C6 and radiolabeled at the 5′-end of the flap with phosphorus-32 (^32^P) (Fig. 3F). Following incubation of the unmodified and the amino acid mimetic-conjugated DNA substrates with recombination FEN1, we ran the samples on denaturing PAGE. FEN1 processed both substrates with comparable efficiency (Fig. 3F), suggesting that small chemical modifications of individual nucleotides on the flap do not affect FEN1’s activity. We, therefore, conclude that a 5′-flap DNA strand with an internal nucleotide cross-linked to an oligopeptide can be processed by FEN1 as long as the oligopeptide is away from the point of annealing and does not sterically block the flap junction. To examine whether a protein of bulky size can hinder FEN1 activity, we further modified our oligonucleotide substrate with biotin and incubated this new substrate (now cy5 labeled) with streptavidin tetramer (60 kDa) to assemble a DPC-like substrate. FEN1 processed this substrate with a much lower efficiency than the C6-modified substrate (Fig. 3G and fig. S3F). This finding is consistent with our observations that cleavage by FEN1 requires the activity of the proteasome or p97/VCP to proteolyze or denature the full-length DPCs and facilitate the tracking and expose the junction.

### FEN1 prevents FA-induced replication stress and chromosomal instability

**Fig. 4.**
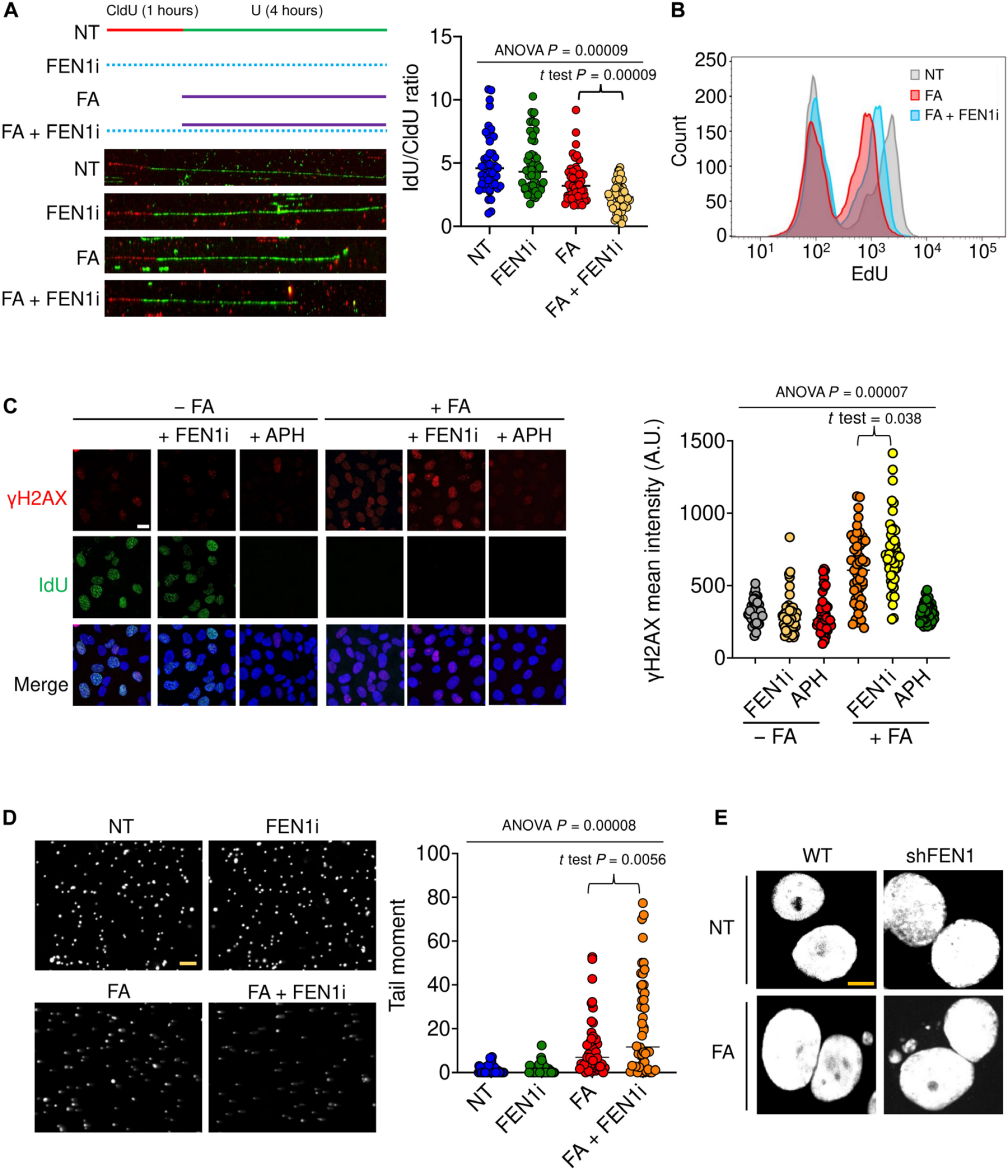
FEN1 prevents FA-induced replication stress and chromosomal instability. (**A**) Top left, labeling protocols for DNA combing assay in U2OS cells. FEN1i, 10 μM; FA, 400 μM. Bottom left, representative images of CldU and IdU tracks from combing assays performed under conditions described in the top panel. Right, IdU/CIdU ratio with means ± SD measured from experiments shown in the left panel. (**B**) EdU incorporation analyzed by flow cytometry. Cells were treated with FA (400 μM) with or without FEN1i (10 μM) for 4 hours and pulsed with EdU (10 μM) for 30 min before harvesting. (**C**) Left, representative images of IF of γH2AX and IdU foci by instant structural illumination microscope. U2OS cells were synchronized in S phase by double thymidine block, followed by IdU incorporation and indicated treatments (400 μM FA, 10 μM FEN1i, 1 μM APH) for 4 hours for IF using anti-γH2AX and anti-BrdU antibodies. Scale bar, 20 μm. Right, quantitation of γH2AX intensity from experiments shown in (E) using ImageJ. *n* = 300 biologically independent cells. (**D**) Left, representative images of neutral comet assay in U2OS cells treated with FEN1i (10 μM, 1 hour), FA (400 μM, 4 hours), and FA + FEN1i (pretreatment with FEN1i for 1 hour then cotreatment for 4 hours). Cells were subjected to neutral comet assay for detection of DNA breaks. Scale bar, 100 μm. Right, quantitation of tail moments from experiments shown on the left using OpenComet, a plugin of ImageJ. *n* = 200 biologically independent cells. (**E**) Representative images of WT and sh*FEN1* interphase cells treated with or without FA. WT and sh*FEN1* MCF-7 cells were exposed to 500 μM FA for 2 hours at 37°C, followed by paraformaldehyde fixation and 4′,6-diamidino-2-phenylindole staining for microscopic analysis of micronuclei.

FA has been implicated in replicative stress and genome instability (*44*). In the absence of FEN1, damaged bases adjoining DPCs are converted into 5′-flaps presumably by the BER pathway but left unrepaired. Upon collision of replication forks, single-ended double-strand breaks (DSBs) are formed (fig. S4A) (*45*). To determine whether FEN1 relieves FA-induced replication stress, we first measured nascent DNA synthesis by DNA combing. Following 1 hour of incorporation of chlorodeoxyuridine (CIdU) in the presence or absence of FEN1 inhibitor FEN1i, iododeoxyuridine (ldU) was added to U2OS cells with or without FA or FEN1 inhibitor for 4 hours before combing analysis. FEN1 catalytic inhibition did not perturb fork progression, as CIdU and IdU track lengths remained unaffected by FEN1 inhibition. By contrast, FA reduced ldU track length, and FA in combination with FEN1 inhibition resulted in a further shortening of the CldU tracks (Fig. 4A). Complementation experiments by expressing FEN1 wild-type (WT) in FEN1 KO cells showed that FEN-WT but not its nuclease-dead mutant D181A rescued IdU track length (fig. S4B), implying alleviation of FA-induced replication by FEN1.

To confirm the impact of FA and FEN1 on DNA replication, we performed fluorescence-activated cell sorting (FACS) to quantify the incorporation of the thymidine analog ethenodeoxyuridine (EdU) during replication. Cells were pretreated with the FEN1 inhibitor, followed by cotreatment with FA for 4 hours and pulse incorporation of EdU for 30 min. Consistent with the DNA combing assays, FEN1 inhibition alone did not affect EdU uptake (fig. S4C). By contrast, FA reduced EdU incorporation, indicative of stalled replication. In line with the combing assay, FA combined with FEN1 inhibition further decreased EdU incorporation (Fig. 4B), whereas rescue expression of FEN1 WT but not of its nuclease dead mutant D181A restored EdU incorporation in FEN1 KO (fig. S4D). Together, these results demonstrate that FEN1 is a critical factor limiting FA-induced replication stress presumably by removing the DPCs generated by FA.

Persistent DPCs formed ahead of replication forks physically obstruct their progression, leading to fork collapse, DSBs, and ultimately chromosomal instability. To test the role of FEN1 in limiting FA-induced DNA damage, we measured γH2AX foci by immunofluorescence (IF) microscopy and found that FA treatment induced γH2AX formation. FA also blocked IdU incorporation, and FEN1 inhibition enhanced FA-induced γH2AX (Fig. 4C). Replication inhibition by aphidicolin (APH) inhibited γH2AX induction by FA, indicating the prominent role of replication for the induction of DNA damage by FA. In parallel, we conducted neutral comet assays in U2OS cells and found that FEN1 inhibition led to an accumulation of FA-induced DNA single-strand breaks (SSBs) and DSBs (Fig. 4D). Yet, OGG1 down-regulation in cells synchronized in S phase prevented the accrual of FA-induced γH2AX (fig. S4E), suggesting that the induction of single-ended DSBs harboring DPCs requires prior BER.

To determine whether FEN1 plays a role in FA-induced chromosomal instability, we assessed micronuclei that arise from lagging acentric chromosome or chromatid fragments in anaphase by microscopy and found an elevation in micronucleus levels in FEN1 small-hairpin RNA (shRNA) KD MCF-7 cells (Fig. 4E and fig. S4, F and G). Together, these data demonstrate a key role of FEN1 in preventing FA-induced replication fork stalling, replicative DNA damage, and chromosomal instability likely by efficiently repairing FA-induced DPCs.

### FEN1 repairs enzymatic TOP2-DPCs independently of the BER pathway

**Fig. 5.**
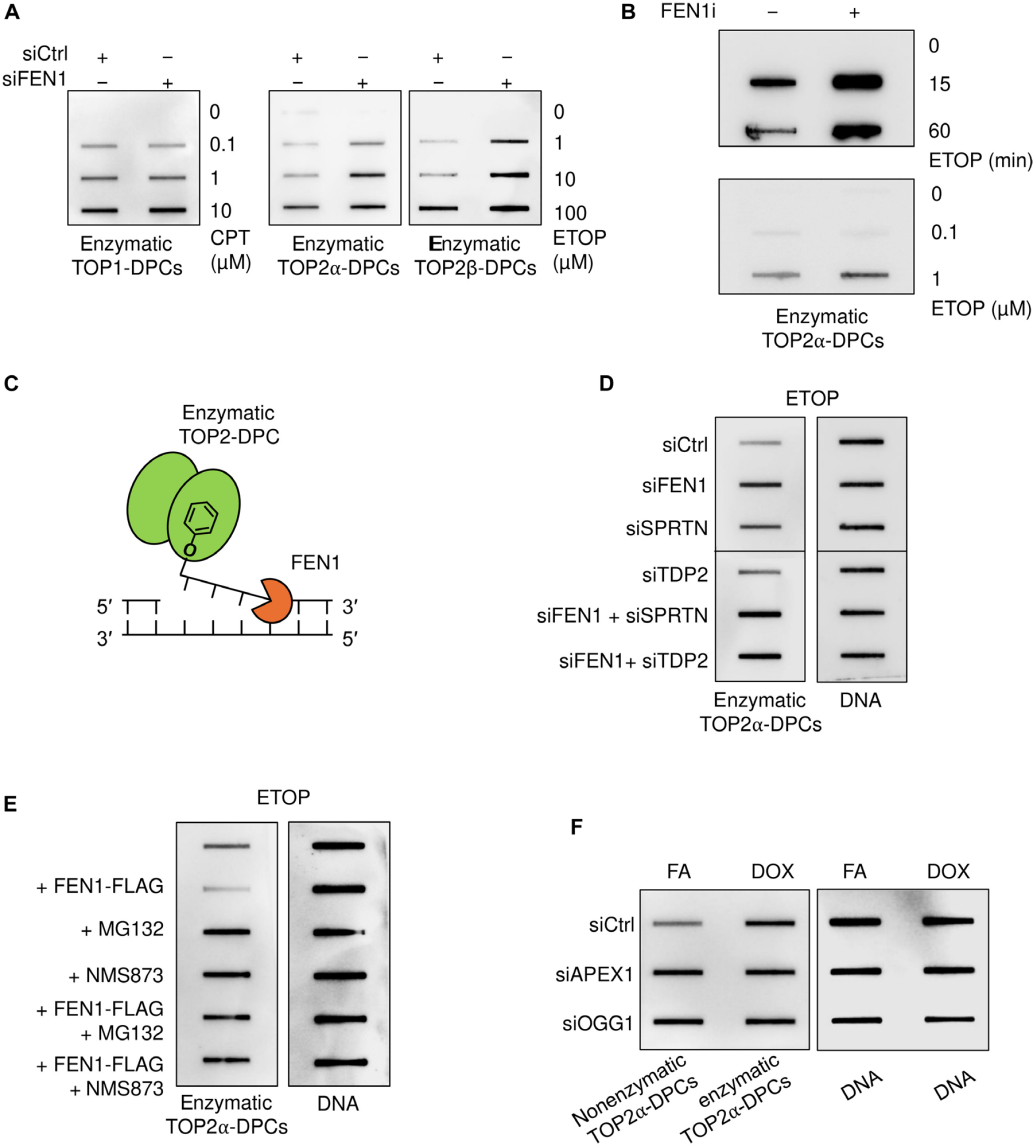
FEN1 repairs enzymatic TOP2-DPCs independently of the BER pathway. (**A**) Representative ICE assays in U2OS cells treated with the indicated agents (CPT, ETOP) for 30 min before immunostaining with the indicated antibodies. (**B**) Representative RADAR assay in HEK293 cells first treated with FEN1 inhibitor FEN1–IN-4 (1 μM, 1 hour) and then cotreated with ETOP at 10 μM for indicated periods (top) or at the indicated concentrations for 1 hour. (**C**) Model depicting the proposed repair of TOP2-DPCs by FEN1. The single-stranded 5′-TOP2-DPCs induced by TOP2 inhibitors such as ETOP or DOX can be converted into 5′ flap structures via posttranslational modifications, conformational changes, or polymerase activities before FEN1 processing. TOP2α-DPCs were probed with TOP2α antibody. (**D**) Representative RADAR assay in HEK293 cells transfected with the indicated siRNAs for 48 hours before treatment with ETOP (10 μM for 1 hour). TOP2α-DPCs were probed with TOP2α antibody, and DNA was probed with anti-DNA antibody. (**E**) Representative RADAR assay in HEK293 cells was first treated with the indicated inhibitors for 1 hour and then cotreated with 10 μM ETOP for 1 hour. (**F**) RADAR assay in HEK293 cells transfected with the indicated siRNAs for 48 hours before 1-hour treatments with FA (1 mM) or DOX (10 μM).

To determine whether FEN1 also repairs enzymatic DPCs, we performed the ICE assays in FEN1-deficient human cells treated with the TOP1 inhibitor camptothecin (CPT) and the TOP2 ETOP to induce enzymatic TOP1 and TOP2-DPCs, two of the best characterized enzymatic DPCs (*19*, *20*). In line with TOP1 generating 3′-DPC cleavage complexes whereas TOP2 forms 5′-DPC cleavage complexes, we found that FEN1 down-regulation increased ETOP-induced TOP2 (both α and β isozymes)–DPCs but not CPT-induced TOP-DPCs (Fig. 5A and fig. S5A). RADAR assays also revealed that cells treated with the FEN1 inhibitor and that the FEN1 KO cells showed higher ETOP-induced TOP2-DPCs than WT cells (Fig. 5B and fig. S5B). These findings were confirmed by showing that the FEN1 inhibitor rendered cells hypersensitive to ETOP (fig. S5C). These data are in accordance with a previous finding that FEN1 has nucleolytic activity against single-stranded 5′-phoshotyrosyl DNA termini (Fig. 5C) (*46*).

DKD of FEN1 and SPRTN and of FEN1 and TDP2, a tyrosine 5-phosphodiesterase both stimulated ETOP-induced TOP2-DPCs in comparison with their respective single knockdown (Fig. 5D and fig. S5D). Further, double knocking down of FEN1 and SPRTN led to hypersensitivity to ETOP (fig. S5E). These data together suggest FEN1 as a salvage pathway in parallel to SPRTN and TDP2. Furthermore, double knockdown of FEN1 and MRE11 also resulted in higher levels of ETOP-induced TOP2-DPCs than the respective single siRNAs, indicating that FEN1 and MRE11 also act as redundant pathways for TOP2-DPC repair (fig. S5F).

Akin to their epistasis in FA-induced DNA repair, both the proteasome and the p97/VCP unfoldase were required for FEN1-mediated processing of the enzymatic TOP2-DPCs (Fig. 5E and fig. S5G). As FEN1-dependent repair of FA-induced nonenzymatic DPCs requires the BER pathway to convert the no-break DPCs into 5′-flap DPCs, we used RADAR assays to test whether FEN1-mediated enzymatic TO2-DPC repair also involves the BER pathway. While down-regulation of APE1 or OGG1 elevated FA-induced nonenzymatic TOP2-DPC levels, inactivation of APE1 or OGG1, on the contrary, did not affect enzymatic TOP2-DPCs induced by doxorubicin (Fig. 5F and fig. S5H), a TOP2 inhibitor that also induces oxidative damage (see Fig. 3, B and C). These findings show that FEN1 can excise enzymatic TOP2-DPCs without the BER pathway.

### PARP1 promotes FEN1-dependent repair of nonenzymatic and enzymatic DPCs

PARP1 trapping or binding to damaged DNA elicits in trans autoPARylation and PARylation of the damaged sites/chromatin and DNA repair proteins, which collectively promote the repair of DNA lesions including SSBs and DSBs by homologous recombination or nonhomologous end-joining, nucleotide excision repair (NER), as well as BER (*31*, *47*). Having established that FEN1 repairs nonenzymatic DPCs and prevents DPC-induced genotoxic events, we sought to identify molecular mechanisms regulating FEN1 in DPC repair.

**Fig. 6.**
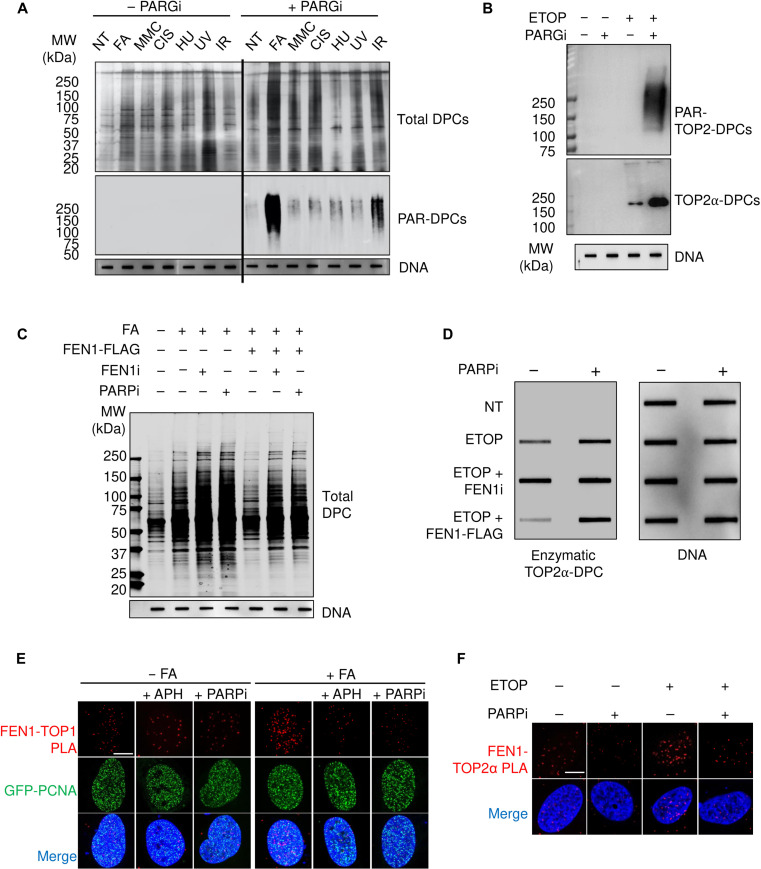
PARP1 induces FEN1-dependent repair of nonenzymatic and enzymatic DPCs. (**A**) The modified RADAR assay in HEK293 cells treated with the indicated DNA damaging agents: FA, 400 μM for 2 hours; hydroxyurea (HU), 1 mM for 2 hours; mitomycin C (MMC), 10 μM for 2 hours; cisplatin, 10 μM for 2 hours; ultraviolet (UV), 20 J/m^2^; infrared (IR), 20 Gy. Cells were exposed to 10 μM PARGi for 1 hour before cotreatments. (**B**) The modified RADAR assay in HEK293 cells. PARGi was used at 10 μM for 1-hour pretreatment and then 1-hour cotreatment with 10 μM ETOP. (**C**) HEK293 cells were transfected with empty vector or FEN1-FLAG overexpression plasmid, followed by the indicated treatments: FA, 400 μM for 2 hours; FEN1i, 10 μM, 1-hour pretreatment +2-hour cotreatment with FA; PARPi, 10 μM, 1-hour pretreatment +2-hour cotreatment with FA. Cells were subjected to the modified RADAR assay for the detection of total DPCs. (**D**) The modified RADAR assay in HEK293 cells transfected with or without FEN1-FLAG expression plasmid for 48 hours before the indicated treatments. PARPi and FEN1i were used at 10 μM for 1-hour pretreatment, followed by cotreatment with ETOP at 10 μM for 1 hour. (**E**) GFP–proliferating cell nuclear antigen (PCNA) expression plasmid-transfected U2OS cells were treated as indicated: FA, 400 μM for 1 hour; APH, 1 μM, 30-min pretreatment +1-hour cotreatment with FA; PARPi, 10 μM, 1-hour pretreatment +1-hour cotreatment with FA). Cells were then subjected to PLA assay to measure TOP1 and FEN1 interaction. Scale bar, 10 μm. (**F**) U2OS cells were treated as indicated: ETOP, 10 μM for 1 hour; PARPi, 10 μM, 1-hour pretreatment +1-hour cotreatment with ETOP). PLA assay was used to measure TOP2α and FEN1. Scale bar, 10 μm.

As our ICE-MS profiling identified PARP1 DPCs among the most abundant DPCs induced by FA (dataset S1) and our RNAi screen found PARP1 as a resistance gene to FA cytotoxicity (see Fig. 2A), we examined the role of PARP1 and PARylation in regulating FEN1-mediated DPC repair. To determine whether FA induction of PARP1-DPCs as well as of other nonenzymatic DPCs activates PARP1 and whether PARylation signaling recruits repair proteins to the DPC sites, we tested whether FA-induced DPCs are PARylated. Using the modified RADAR assays in HEK293 cells treated with FA along with several other DPC inducers, we initially failed to detect any PARylation signal on these DPCs. However, pretreatment with PARG inhibitor (*48*–*50*) led to readily detectable PARylation of the DPCs induced by FA (Fig. 6A and fig. S6A). We also observed upshifting of the DPC signal (indicative of PARylated DPCs of higher molecular weight and hence lower electrophoretic mobility) (Fig. 6A). These results demonstrate that FA-induced DPCs are specifically and reversibly PARylated. Akin to FA-induced nonenzymatic DPCs, enzymatic TOP2-DPCs induced by ETOP appeared to be PARylated and promptly reversed by PARG, as evidenced by the fact that TOP2-DPC PARylation was only detectable when PARG was blocked (Fig. 6B).

Next, we assessed whether FEN1 can process PARylated DPCs. Incubating RADAR samples from cells exposed to FA in the presence of PARG inhibitor with recombinant FEN1 showed that FEN1 remained able to release PARylated DPCs induced by FA (fig. S6B). This result shows that PARylation does not interfere with FEN1 activity and suggests that PARylation of the DPCs serves as a recruitment signal, as PAR-branched polymers resemble nucleic acid molecules.

To elucidate the role of PARP1 in nonenzymatic DPC repair, we performed modified RADAR assays in HEK293 cells transfected with FEN1 plasmid and observed that FEN1 overexpression facilitates the removal of FA-induced DPCs—an effect that was suppressed by FEN1 and PARP1 inhibitors (Fig. 6C and fig. S6C). We also found that enzymatic TOP2-DPCs induced by ETOP were cleared by up-regulation of FEN1 and that the FEN1-dependent removal of ETOP-induced TOP2-DPCs was inhibited by the potent and specific PARP inhibitor, talazoparib (Fig. 6D and fig. S6D). Further, PLA assays showed that PARP1 inhibition diminished FA-induced TOP1 and FEN1 interaction as did APH (Fig. 6E and fig. S6, E and F). Similarly, ETOP-induced TOP2α-FEN1 colocalization was disrupted by PARP1 inhibition (Fig. 6F and fig. S6, G and H). Together, these data demonstrate the enabling role of PARP1 and PARylation in FEN1-mediated DPC repair.

### ADP-ribosylation of FEN1 at glutamic acid residue 285 recruits FEN1 to DPCs

**Fig. 7.**
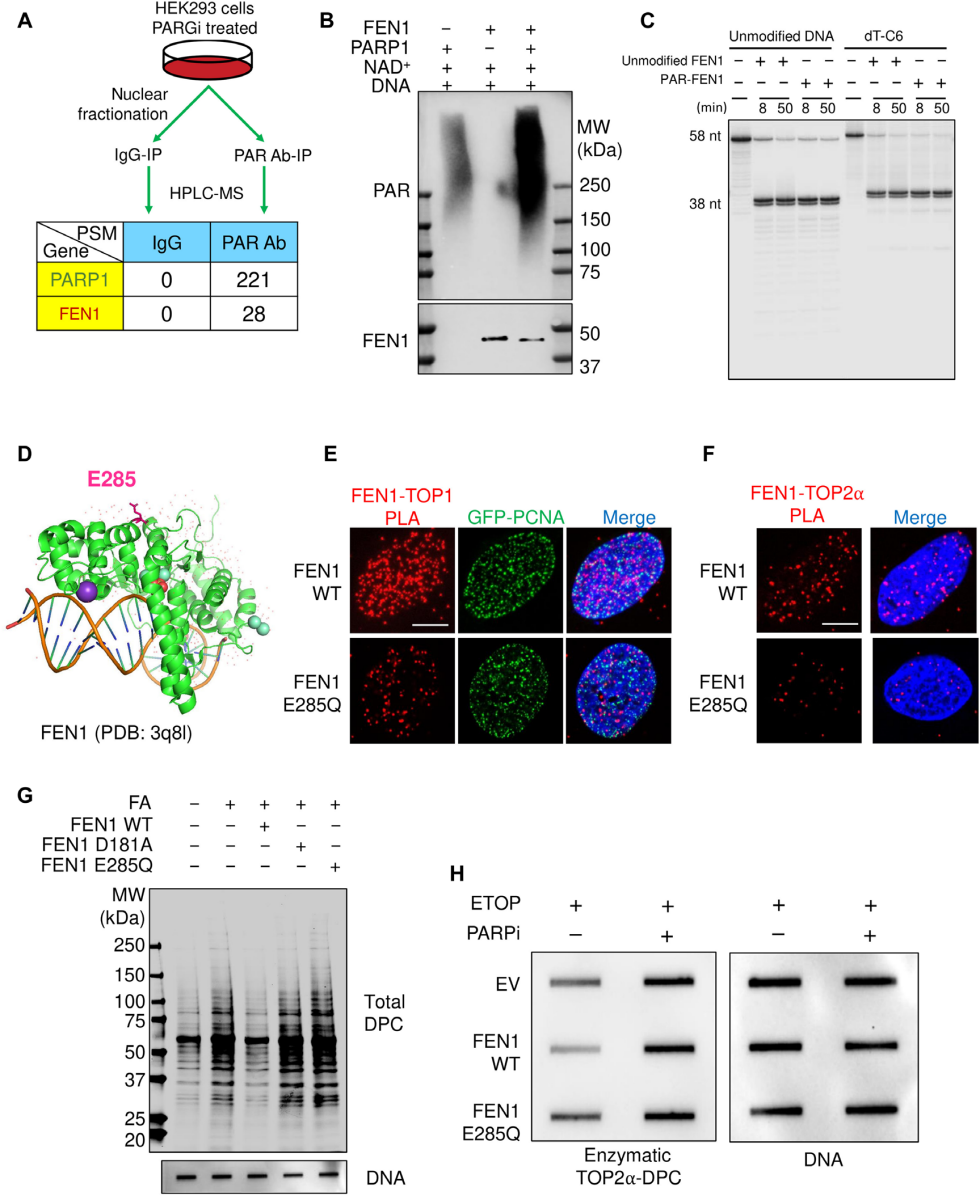
ADP-ribosylation of FEN1 at glutamic acid 285 residue localizes FEN1 to DPC sites for repair. (**A**) Experimental setup for PAR antibody-IP-MS in HEK293 cells. PSM, peptide spectrum match. (**B**) Representative in vitro PARylation assay with recombinant FEN1 protein, recombinant PARP1 protein, NAD^+^, and activated DNA. Following 20-min incubation at room temperature, the samples were subjected to Western blotting using anti-PAR and anti-FEN1 antibodies. Anti-FEN1 antibody failed to detect PARylated FEN1 likely because PAR polymers blocked the epitope. (**C**) Representative activity assay testing unmodified and PARylated recombinant FEN1 toward unmodified and C6-modified DNA substrates for the indicated times. ^32^P labeled DNA products following the activity assay were visualized by PAGE electrophoresis. (**D**) Structure of FEN1 with DNA substrate, SM^3+^ and K^+^ (PDB: 3q8l) highlighting glutamic acid residue 285 (E285), an ADP-ribosylation site identified by ELTA-MS. (**E**) GFP-PCNA expressing U2OS cells transfected with FEN1-FLAG WT or FEN1-FLAG E285Q expression plasmid were treated with 400 μM FA for 1 hour. Cells were then subjected to PLA assay to measure TOP1 and FEN1-FLAG interaction using their TOP1 and FLAG antibodies. Scale bar, 10 μm. (**F**) U2OS cells transfected with FEN1-FLAG WT or FEN1-FLAG E285Q expression plasmid were treated with 10 μM ETOP for 1 hour. Cells were then subjected to PLA assay to measure TOP2α and FEN1-FLAG interaction using TOP2α and FLAG antibodies. Scale bar, 10 μm. (**G**) HEK293 cells were transfected with empty vector or FEN1-FLAG overexpression plasmid, followed by treatment with 400 μM FA for 2 hours. Cells were subjected to the modified RADAR assay for detection of total DPCs by Coomassie stain. (**H**) HEK293 cells were transfected with empty vector or indicated FEN1-FLAG overexpression plasmid, followed by treatment with 10 μM ETOP for 30 min. Cells were subjected to the RADAR assay for detection of enzymatic TOP2α-DPCs and DNA using their antibodies.

To determine whether FEN1 is a direct substrate of PARP1, we conducted PAR antibody–based nuclear immunoprecipitation (IP) under denaturing conditions and performed MS analyses in HEK293 cells treated with PARG inhibitor (Fig. 7A and dataset S4). We identified FEN1 as a PARylation substrate and confirmed this result by in vitro PARylation assays using recombinant FEN1 and PARP1 proteins (Fig. 7B). Further, FLAG-IP in HEK293 cells transfected with FLAG-FEN1 stimulated FEN1 PARylation by FA, and PARG inhibition further enhanced FA-stimulated FEN1 PARylation (fig. S7A).

We next performed in vitro PARylation assays with recombinant FEN1 and tested the activity of PARylated FEN1 using the 5′-flap DNA substrate (see Fig. 3F). Unmodified and PARylated FEN1 exhibited comparable efficiency both with the unmodified and amino modifier C6-conjugated substrates (Fig. 7C). These experiments show that PARylation of FEN1 does not affect the catalytic activity of FEN1 toward to 5′-flap DPCs and suggest that PARylation of FEN1 may contribute to its recruitment to FA-induced DPCs.

To test the hypothesis that FEN1 PARylation by PARP1 drives FEN1 to DPCs, we applied the ELTA (enzymatic labeling of terminal ADP-ribose) method for MS identification of the ADP-ribosylation sites on FEN1 (*51*). In brief, we first PARylated recombinant FEN1 in vitro using PARP1 and nicotinamide adenine dinucleotide (oxidized form) (NAD^+^). Following trypsin digestion, we labeled FEN1 peptide–conjugated ADP-ribose polymers at their 2′-OH termini using the enzyme OAS1 and deoxyadenosine triphosphate (dATP). After enrichment of the labeled FEN1 peptide–ADP–ribose conjugates, we treated the samples with NudT16, which cleaves the pyrophosphate bond within ADP-ribose to leave a phosphoribosyl group on protein residues that were previously ADP-ribosylated before performing MS profiling of the ADP-ribosylation sites. With this method, we identified glutamic acid residue 285 (E285) as a major ADP-ribosylation site of FEN1 (Fig. 7D). By mutating this residue to glutamine (E285Q) and performing in vitro PARylation assays, we corroborated our findings by observing that the FEN1 mutant E285Q exhibited lower levels of PARylation than its WT counterpart (fig. S7B). In vitro activity assay also showed that the E285Q mutant did not affect its activity against the streptavidin-biotin–modified 5′ flap DNA substrate (fig. S7C).

To characterize the functional role of the E285 PARylation of FEN1, we conducted PLA assays and found that mutating E285 led to a reduction in the level of FA-induced TOP1-FEN1 and ETOP-induced TOP2α-FEN1 interaction foci (Fig. 7, E and F, and fig. S7, D and E). As protein PARylation is promptly reversed by the PARG to enable the downstream repair events signaled by PARylation, the highly transient PARylation of FEN1 does not affect its retention at DPCs. We therefore rule out the possibility that the E285 PARylation affects FEN1 retention at DPCs and conclude that it likely acts as a recruitment mechanism for the localization of FEN1 to DPCs. Accordingly, modified RADAR assays showed that up-regulation FEN1 E285Q failed to promote the removal of FA-induced DPC (Fig. 7G and fig. S7F) and to attenuate FA-induced replication stress as opposed to the native FEN1 counterpart (fig. S7G). FEN1 E285Q was also unable to remove ETOP-induced enzymatic TOP2α-DPCs (Fig. 7H and fig. S7H).

By carrying out clonogenic assays, we found that rescue expression of FEN1 WT but not E285Q desensitized FEN1 KO cells to FA and ETOP (fig. S7, I and J), respectively, and that FEN1 WT expression failed to rescue FA- or ETOP-induced FEN1 KO cell death in the presence of PARPi (fig. S7, I and J). In sum, from these findings, we conclude that PARP1 activation by the DPC-conjugated 5′-flaps PARylates FEN1 to mediate FEN1 localization to the DPCs and its subsequent endonuclease activity toward 5′-flap DPCs.

## DISCUSSION

As opposed to enzymatic DPCs such as the topoisomerase DPCs that are linked to the ends of the nucleic acid broken by the enzymes, FA cross-links proteins to DNA without introducing terminal DNA breaks, raising the possibility that repair nuclease (s) must incise the DNA backbone adjacent to the DPCs (*17*, *19*). In the present study, we systematically profiled proteins cross-linked to DNA by FA using ICE-MS and identified FEN1 as a previously unrecognized nuclease excising FA-induced DPCs downstream from a PARylation pathway.

**Fig. 8.**
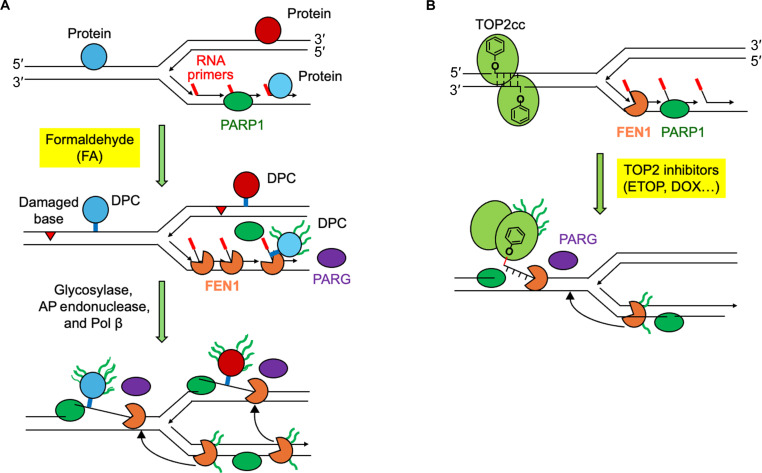
Working models depict the role of PARylation in nonenzymatic and enzymatic DPC repair by FEN1. (**A**) DPCs formed within the 5′-flap of Okazaki fragments during strand displacement are sensed by PARP1 leading to PARylation, which activates FEN1 for repair following dePARylation of the DPCs by PARG. Concurrent damaged bases adjacent to non-end DPCs (in the front of the fork and on the leading strand behind the fork) are converted into DPC-harboring 5′-flaps by the BER proteins with SSBs that can be detected by PARP1 for PARylation to signal FEN1. FEN1 enriched near Okazaki fragments are in PARylated by PARP1 for their translocation to the front of fork and to the leading strand behind the fork to cleave DPC-harboring 5′-flaps following dePARylation by PARG. (**B**) TOP2α are homodimeric enzymes that can act in front of the replication fork to relax accumulated positive supercoils. The homodimers cut both strands of DNA and pass another DNA molecule through the break. The 5′ end of each break is covalently linked to the tyrosine in the active center of each of the two subunits of the protein (TOP2αcc). In this configuration, the two sides of the nicked DNA are held together by the strong protein-protein interactions between the two subunits of TOP2, allowing the nicks to be faithfully resealed in situ. These transient enzyme-DNA intermediate can be trapped by their inhibitors such as ETOP and DOX. The binding of the inhibitor to the interface between one of the subunits and DNA leads to the formation of a single-stranded 5′ TOP2-DPC, which can be converted to a 5′ flap structure via posttranslational modifications, conformational changes, or polymerase activities. PARP1 senses the TOP2-linked SSB and PARylates the DPC to signal FEN1. FEN1 at Okazaki fragments at the rear of the fork is also PARylated by PARP1, which drives FEN1 to the DPC site for cleavage upon dePARylation by PARG.

FEN1 plays crucial roles in DNA replication and repair by processing 5′-flap structures that arise from strand displacement synthesis by Pol δ during Okazaki fragment maturation and from displacement of DNA broken ends by Pol β during long-patch BER (*28*, *52*). On the basis of the roles of FEN1 in DNA replication and repair, we propose that FEN1 processes DPCs formed behind the forks on the flap of Okazaki fragments (lagging strand) or ahead of replication forks on the leading strand (or its parental strand) (Fig. 8A). For FEN1 to excise DPCs in front of the forks, we hypothesized that 5′-flap structures are formed as by-products of BER processing of damaged bases induced by FA in close proximity to the DPCs. Accordingly, we observed induction of base damage by FA colocalizing with DPCs, supporting our abovementioned model for FEN1-mediated repair of DPCs formed ahead of the replication fork (Fig. 8A). As a result, we found that due to its endonuclease activity, FEN1 attenuates FA-induced replication stress to maintain DNA synthesis.

We also found that FEN1 repairs enzymatic TOP2-DPCs independently of the BER pathway (Fig. 8B), as opposed to its double role in the repair of FA-induced nonenzymatic DPCs. Although TOP2 homodimers cleave both strands of the DNA duplex to generate the enzyme-DNA covalent intermediates (TOP2 cleavage complexes or TOP2ccs) and rejoin the broken ends after strand passage, rejoining of the two strands can be independently inhibited by TOP2 inhibitors (*53*). A large fraction of DNA breaks induced by the clinical TOP2 inhibitor ETOP are protein-linked DNA SSBs (*54*–*56*). These 5′-single-strand DPCs can be converted into 5′-flap structures presumably through conformational alteration in the protein or polymerase activity without the BER pathway as a precondition for its cleavage (Fig. 8B).

An earlier study showed that perturbation of replication by down-regulation of FEN1 enhances PARP activity (*49*), and our study further demonstrates that PARP1-mediated ADP-ribosylation recruits FEN1 to DPCs. We found that both FA-induced nonenzymatic DPCs and ETOP-induced enzymatic TOP2-DPCs as well as FEN1 are PARylated and that PARylation is promptly reversed by PARG and hence highly transient. We propose that DPC PARylation acts as a signal to recruit FEN1 with PARylation acting as an interaction scaffold and that PARylation of FEN1 further recruits FEN1 to the PARylated DPCs (Fig. 8, A and B). Such a model is supported by our finding that FEN1 E285 PARylation is required for its colocalization with DPCs such as TOP1 and TOP2α (*7*, *8*). Given that FEN1 is enriched near Okazaki fragments for their maturation and that DPCs formed within the flap of Okazaki fragments can be easily detected by PARP1 and cleaved by FEN1, it is plausible that PARylation drives the translocation of FEN1 from the rear of the fork to its front or to the leading strand behind the fork to repair DPCs formed within those two regions (Fig. 8A).

It is generally established that proteolytic digestion of enzymatic DPCs (e.g., TOP-DPCs) enables or facilitates the processing of the phosphotyrosyl bonds between the enzymes and DNA by tyrosyl-DNA phosphodiesterases (*19*, *20*). This is also the case for FEN1-mediated repair of nonenzymatic DPCs and enzymatic TOP2-DPCs, as demonstrated by our observation that down-regulation of FEN1 and SPRTN markedly enhanced the accumulation of both the nonenzymatic DPCs induced by FA and the enzymatic TOP2-DPCs induced by ETOP in comparison with their respective single down-regulation. In addition, FEN1 single knockdown was found to elevate full-length nonenzymatic DPCs. If partial proteolysis of the DPCs by either SPRTN or the proteasome was a precondition for FEN1-dependent cleavage of DPCs, partially proteolyzed DPCs instead of the full-length counterpart should have accumulated upon FEN1 single knocking down. Thus, our findings directly suggest that FEN1 and the SPRTN protease serve as parallel pathways for both nonenzymatic and enzymatic DPC repair. The partially proteolyzed enzymatic TOP2 DPCs are repaired by TDP2 (*19*, *20*), the 5′-tyrosine-DNA phosphodiesterase, whereas FA-induced DPCs might be repaired by other nucleolytic mechanisms or by homologous recombination during replication following their proteolysis. Yet, how transcription-associated DPCs are repaired remains to be clarified. Our preliminary finding that down-regulation of XPF (ERCC4) or XPG (ERCC5) conferred hypersensitivity to FA and accumulated FA-induced DPCs in the cell suggests an involvement of transcription-coupled NER in the repair of general nonenzymatic DPCs and hence warrants further investigation.

In conclusion, our work found a nucleolytic pathway as a repair mechanism for nonenzymatic and enzymatic DPCs and a central factor for avoiding DPC-induced genome instability. Defects in (both enzymatic and nonenzymatic) DPC repair have been implicated as a major cause of progeroid features, cancers including hepatocellular carcinoma, neurodegeneration, as well as immunodeficiencies (*6*, *19*). Consistently, somatic FEN1 mutations can lead to autoimmunity, chronic inflammation, and cancers (*28*, *57*). Our work provides previously unrecognized pieces to the puzzle of DPC repair and a molecular foundation for the etiology of DPC-induced diseases.

## MATERIALS AND METHODS

### Human cell culture

HEK293 cells, human breast cancer MCF7 cells, and human bone osteosarcoma U2OS cells were in cultured in Dulbecco’s modified Eagle’s medium (DMEM) (Life Technologies) supplemented with 10% (v/v) fetal bovine serum, penicillin (100 U/ml), streptomycin (100 μg/ml), and 1× GlutaMax in tissue culture dishes at 37°C in a humidified CO_2_–regulated (5%) incubator.

### Chemicals

The chemicals used are as follows: FA solution (Sigma-Aldrich), FEN1-IN-4 (FEN1i, Selleck), PDD 00017273 (PARGi, Tocris), the replication inhibitor APH (Sigma-Aldrich), ETOP (Sigma-Aldrich), talazoparib (PARPi, Selleck), AcquaStain protein gel Coomassie stain (Bulldog Bio), and Silver Stain solutions (Bio-Rad).

### Antibodies

The following antibodies were used: anti-PAR, mouse monoclonal, Trevigen, 4335-MC-100; anti-PAR (10H), mouse monoclonal, Enzo Life Sciences, ALX-804-220; anti-TOP1, mouse monoclonal, BD Biosciences, 556597; anti–double-stranded DNA (dsDNA), mouse monoclonal, Abcam, ab27156; anti-FLAG, mouse monoclonal, Sigma-Aldrich, F1804; anti-FLAG, rabbit polyclonal, Sigma-Aldrich, F7425; anti-FEN1, rabbit polyclonal, Cell Signaling Technology, 2746; anti-XPF, rabbit monoclonal, 13465; anti-XPG, mouse monoclonal, Santa Cruz Biotechnology, 13563; anti-APE1, rabbit monoclonal, Cell Signaling Technology, 10519; anti-EXO1, rabbit polyclonal, Abcam, 95068, anti-DNA2, rabbit polyclonal, Abcam, 96488; anti-MRE11, mouse monoclonal, GeneTex, 70212; anti-TDP1, rabbit polyclonal, Bethyl Laboratories, A301-618A; anti-TDP2, mouse monoclonal, Santa Cruz Biotechnology, 377280; anti-PARP1 (F2), mouse monoclonal, Santa Cruz Biotechnology, sc-8007; anti-PARG, rabbit monoclonal, Cell Signaling Technology, 66564; and anti-γH2AX, rabbit polyclonal, Cell Signaling Technology, 66564; anti-BrdU (5-bromo-2′-deoxyuridine), mouse monoclonal, ab8152.

### Recombinant proteins

Human recombinant FEN1 is a gift from R. Prasad at NIEHS. Human recombinant PARP1 was purified from *Escherichia coli* as described (*58*). Human 2′ − 5′-oligoadenylate synthase 1 (OAS1) was purified from SF9 insect cells as described.

### Expression plasmids

The following expression plasmids were used: pShuttle-FLAG-FEN1, Addgene, 35027; pCMV-6 × His-TOP1-HaloTag (*17*); pCMV PARP1-3 × Flag, Addgene, 111575; pCMV6-AC-FEN1-turboGFP, OriGene, RG201785; and pCMV6-AC-PCNA-turboGFP, OriGene, RG201741. Forty-eight–hour transfection was performed using Lipofectamine 3000 transfection reagent (Invitrogen) following the manufacturer’s instructions.

### siRNA and shRNA

The following expression plasmids were used: control siRNA, Dharmacon, D-001206-13-05; FEN1 siRNA, Dharmacon, M-010344-01; ERCC4 (XPF) siRNA, Dharmacon, M-019946-00; ERCC5 (XPG) siRNA, Dharmacon, M-006626-01; APEX1 (APE1) siRNA, Dharmacon, M-010237-01; DNA2 siRNA, Dharmacon, M-026431-01; EXO1 siRNA, Dharmacon, M-013120-00; MRE11 siRNA, Dharmacon, M-009271-01; TDP1 siRNA, Dharmacon, M-016112-01; and TDP2 siRNA, Dharma, M-017578-00. Seventy-two–hour transfection was performed using Lipofectamine RNAiMAX transfection reagent (Invitrogen) following the manufacturer’s instructions.

### Lentivirus-mediated gene silencing of human FEN1

shRNA oligonucleotides targeting FEN1 (shown in the following table) were cloned into pLKO.1 (catalog no. 8453, Addgene) digested with EcoRI and AgeI. pLKO.1-shFEN1 or pLKO.1 control vector was simultaneously transfected into LentiX293T (catalog no. 632180 Clontech, Japan) with packaging plasmid, pSPAX2 (catalog no. 12260, Addgene) and envelop plasmid, pMD2.G (catalog no. 12259, Addgene). After harvesting the medium (3 ml) containing lentiviral particles, we enriched the lentiviral particles with Lent-X Concentrator (catalog no. 631231, Clontech, Japan) according to the manufacturer’s protocol. The supernatant-containing virus was mixed with WT MCF-7 cells. The infected cells (Puromycin resistant) were enriched by puromycin drug selection for 72 hours. Down-regulation of FEN1 expression was confirmed by Western blotting using anti-FEN1 antibody.

Oligonucleotides (shRNA sequences for pLKO.1 vector)

FEN1, forward primer,

5’-CCGGGATGCCTCTATGAGCATTTATCTCGAGATAAATGCTCATA

GAGGCATCTTTTTG -3’

FEN1, reverse primer,

5’-AATTCAAAAAGATGCCTCTATGAGCATTTATCTCGAGATAAAT

GCTCATAGAGGCATC -3′

### CRISRP knocking out of human FEN1

A pool of three different gRNA plasmids was purchased from Santa Cruz Biotechnology (sc-403168):

5′-AGCCCGCCGCTCACTGCGTT-3′;

5′-GCCGTTCTCCATCATGCGAA-3′;

5′-CTACCGCACCATTCGCATGA-3′.

The plasmids were transfected with Lipofectamine 3000 in HT29 cells. The transfected cells were enriched by a BD FACSAria IIu cell sorter for green fluorescent protein (GFP) sorting before isolation of single clones and screening for loss of FEN1B by Western blotting.

### RNAi high-throughput screening

A focused screen was performed using the Dharmacon ON-TARGETplus Human DNA Damage Response siRNA library (Horizon Discovery, GU-106005-025) targeting 239 genes with 4 distinct siRNAs per target, 1 siRNA per well for a total of 956 siRNAs arrayed into three 384-well plates. MCF7 cells (750 cells per well) were reverse transfected using Lipofectamine RNAiMAX (0.15 μl per well, Thermo Fisher Scientific, 13778150) with a final siRNA concentration of 20 nM. Two sets of screens were performed in parallel. Twenty-four hours after transfection, one set was treated with 80 µM FA and the other with vehicle dimethyl sulfoxide (DMSO), for an additional 3 days. On day 4, the cell numbers were evaluated using CellTiter-Glo One luminescence (Promega, G8462) read on a BMG Pherastar FSX plate reader.

Screen performance was evaluated using AllStars Hs cell death control siRNA (Qiagen) compared to nontargeting control siRNA#2 (Thermo Fisher Scientific). Screen z’ averaged 0.7, indicating excellent screen performance. Ratios of luminescence of FA-treated wells normalized to median luminescence of FA-treated non-targeting (NT) siRNA wells over luminescence of vehicle-treated wells normalized to median luminescence of vehicle-treated NT siRNA wells were calculated to determine the combined effect of siRNA knockdown and FA treatment. These ratios were transformed to *z* scores using mean (μ) and SD (σ), *z* = (*x*−μ)/σ. Genes with negative median *z* score of the four siRNAs were considered to be targets whose knockdown sensitized cells to FA-induced damage.

### Western blotting

Cellular proteins were detected by lysing cells with radioimmunoprecipitation assay (RIPA) buffer [150 mM NaCl, 1% NP-40, 0.5% sodium deoxycholate, 0.1% SDS, 50 mM tris (pH 7.5), 1 mM dithiothreitol (DTT), and protease inhibitor cocktail], followed by sonication and centrifugation. The supernatant was collected and boiled for 10 min, analyzed by SDS-PAGE, and immunoblotted with various antibodies as indicated. TOP1 down-regulation was monitored using alkaline lysis method as described previously.

### Viability assay

A total of 10,000 cells were seeded in 96-well white plates (PerkinElmer Life Sciences, 6007680) in triplicate in 100 μl of medium per well overnight. The next day, the cells were exposed to drugs and incubated for 72 hours. Cellular viability was determined using the ATPlite 1-step kits (PerkinElmer). Fifty microliters of ATPlite solution was added in 96-well plates for 15 min, followed by luminescence measurement with an EnVision 2104 Multilabel Reader (PerkinElmer). The ATP level in untreated cells was defined as 100%. Viability (%) of treated cells was defined as ATP-treated cells/ATP-untreated cells × 100.

### In vitro FEN1 PARylation

Recombinant FEN1 (5 μg) was incubated with 200 nM recombinant PARP1 enzyme (5 μg) in 1 × PARylation buffer [50 mM tris-HCl (pH 8.0), 50 mM NaCl, 10 mM MgCl_2_, 2% glycerol, and 1 mM DTT] and activated DNA (BPS Bioscience). One micromolar NAD^+^ was added to the reaction as indicated. The reactions were incubated at room temperature for 20 min and inactivated by adding talazoparib (1 μM) for ELTA-MS analysis or by adding SDS sample buffer for Western blotting analysis.

### FEN1 activity assay

The following oligonucleotides were used in the study: D_amino_ (downstream primer that forms a 38 nt flap)

5′-TCGCGCGTTTCACGCCTGTTACTTAATT*CACTGGCCGTCGTTTTACAACGTGACTGGG.

* The 5-methyl group in thymine of T28 is modified with the amino modifier C6.

U1 (upstream primer)

5′-CGCCAGGGTTTTCCCAGTCACGACC

T_amino_ (template)

5′-GCCCAGTCACGTTGTAAAACGGGTCGTGACTGGGAAAACCCTGGCG

The 3′-end regions of the downstream primer D_amino_ is homologous with the 5′-end of its template. Once annealed, these primers create substrates with unannealed 5′-tails as shown in the figures. The upstream primer U1 was annealed to its template to create a nick at the base of the unannealed 5′-tail of the downstream primer D_amino_. Before annealing, the downstream primer D_amino_ was either 5′-phosphorylated and radiolabeled using [γ-32P]ATP (PerkinElmer) and T4 polynucleotide kinase (NEB) following the NEB online protocol or 5′ labeled with Cy5 dye. D_amino_ was then purified with a mini-Quick Spin DNA Columns (Roche) to remove unincorporated ATP-γ-32P, followed by annealing of the oligos.

FEN1 with or without PARylation (5 nM) was mixed with the unmodified and dT-C6–modified DNA substrates (10 nM) in 20 μl of reaction buffer containing 20 mM tris-HCl (pH 7.4), 40 mM potassium chloride, 5 mM MgCl_2_, bovine serum albumin (BSA, 0.1 mg/ml), and 2 mM DTT. After being incubated at room temperature for an indicated period of time, cleavage products (20 μl) were mixed with 20 μl 2 × formamide gel–loading buffer (10 mM EDTA, 0.025% bromophenol blue, 0.025% xylene cyanol FF, and 0.2% SDS dissolved in formamide), heat denatured at 95°C for 3 min, and separated on an 18% acrylamide gel containing 7 M urea. Gel was then dried and imaged with a GE Typhoon Phosphorimager.

### Proximity ligation assay

Duolink PLA fluorescence assay (Sigma-Aldrich, catalog no. DUO92101) was performed following the manufacturer’s instructions. In brief, U2OS cells were seeded on coverslips and treated with CPT for 30 min. After treatment, the cells were washed with 1 × phosphate-buffered saline (PBS) and fixed for 15 min at 4°C in 4% paraformaldehyde (PFA) in PBS and permeabilized with 0.25% Triton X-100 in PBS for 15 min at 4°C. The coverslips were blocked with Duolink blocking solution and incubated with indicated antibodies in the Duolink antibody diluent overnight, followed by incubation with PLUS and MINUS PLA probes, ligation, and amplification. The coverslips were then washed and mounted using mounting medium with 4′,6-diamidino-2-phenylindole (DAPI). Images were captured on a wide-field microscope, processed using ImageJ, and analyzed using Imaris.

### Detection of DPCs and their ADP-ribosylation

After FA treatment, 1 × 10^6^ human cells in 35-mm dish per sample were washed with 1 × PBS and lysed with 600 μl of DNAzol (Invitrogen), followed by precipitation with 300 μl of 200 proof ethanol. The nucleic acids were collected, washed with 75% ethanol, resuspended in 200 μl of Tris-EDTA (TE) buffer, and then heated at 65°C for 15 min, followed by shearing with sonication (40% output for 10-s pulse and 10-s rest for four times). The samples were centrifuged at 15,000 rpm for 5 min at 4°C, and the supernatants were collected. One microliter of the sample was removed for spectrophotometric measurement of absorbance at 260 nm to quantitate DNA content (NanoDrop). Ten micrograms of DNA from each sample was digested with 50 U of micrococcal nuclease (100 U/μl, Thermo Fisher Scientific) in the presence of 5 mM CaCl_2_, followed by gel electrophoresis on 4 to 15% precast polyacrylamide gel (Bio-Rad) for detection of total DPCs using Coomassie stain or silver stain and PARylated DPCs using anti-PAR antibody. Because of the extremely low abundance of PARylated DPCs, the samples were run in parallel gels to detect total and PARylated DPCs separately instead of stripping and reprobing the same membrane for their detection. In addition, 2 μg of each sample was subjected to slot-blot for immunoblotting with anti-dsDNA antibody as a loading control to verify that amounts of DNA were digested with micrococcal nuclease.

### ICE assay and MS profiling

Cells were exposed to 400 μM for 2 hours and then lysed in sarkosyl solution (1% w/v). The lysates were sheared with a 25-gauge 5/8 needle and loaded onto CsCl solution (150% w/v) for ultracentrifugation in NVT 65.2 rotor (Beckman coulter) at 42,000 rpm for 20 hours at 4°C. The resulting nucleic acid pellets were retrieved and suspended in TE buffer. For MS analysis, ICE samples were treated with ribonucleases A and T1 mix to eliminate RNA contamination, followed by addition of 1/10 volume of 3 M sodium acetate sodium acetate and 2.5 volume of 200 proof ethanol. After 20 min of full-speed centrifugation, the resulting DPC-containing DNA samples were retrieved, resuspended in ddH_2_O, and digested with micrococcal nuclease (100 U per sample) for 1 hour at room temperature to release the cross-linked proteins. Released protein samples were in-solution digested with trypsin following the filter-aided sample preparation protocol. Dried peptides were solubilized in 2% acetonitrile, 0.5% acetic acid, and 97.5% water for MS analysis. They were trapped on a trapping column and separated on a 75 μm by 15 cm, 2-μm Acclaim PepMap reverse phase column (Thermo Fisher Scientific) using an UltiMate 3000 RSLCnano HPLC (Thermo Fisher Scientific). The peptides were separated at a flow rate of 300 nl/min followed by online analysis by tandem MS using a Thermo Orbitrap Fusion mass spectrometer. The peptides were eluted into the mass spectrometer using a linear gradient from 96% mobile phase A (0.1% formic acid in water) to 55% mobile phase B (0.1% formic acid in acetonitrile). Parent full-scan mass spectra were collected in the Orbitrap mass analyzer set to acquire data at 120,000 full width at half maximum resolution; ions were then isolated in the quadrupole mass filter, fragmented within the HCD (higher-energy collisional dissociation) cell (HCD normalized energy of 32%; stepped, ±3%), and the product ions were analyzed in the ion trap. Proteome Discoverer 2.2 (Thermo Fisher Scientific) was used to search the data against human proteins from the UniProt database using SequestHT. The search was limited to tryptic peptides, with maximally two missed cleavages allowed. Cysteine carbamidomethylation was set as a fixed modification, and methionine oxidation was set as a variable modification. Diglycine modification to lysine was set as a variable modification for experiments to identify sites of enzymatic post-translational modifications (PTMs). The precursor mass tolerance was 10 parts per million, and the fragment mass tolerance was 0.6 Da. The Percolator node was used to score and rank peptide matches using a 1% false discovery rate.

### Detection and quantitation of 8-OXO-dG and AP sites

DNA form HEK293 cells were prepared using RADAR assay following treatment with agents FA, campothecin or doxorubicin. Detection and quantitation of 8-OXO-dG and apurinic or apyrimidinic (AP or abasic) sites in the samples were performed using HT 8-oxo-dG enzyme-linked immunosorbent assay kit (R&D Systems) and AP Sites Quantitation Kit (Cell Biolabs), respectively, following the manufacturer’s instruction. Analyses were conducted using GraphPad Prism.

### FLAG IP

The cells were washed with 1 × PBS and lysed in 200 μl of IP lysis buffer [5 mM tris-HCl (pH 7.4), 150 mM NaCl, 1 mM EDTA, 1% NP-40, 0.2% Triton X-100, 5% glycerol, 1 mM DTT, 20 mM *N*-ethylmaleimide (Sigma-Aldrich), and protease inhibitor cocktail] on a shaker for 15 min at 4°C, followed by sonication and centrifugation. The supernatant was collected and treated with 1 μl of benzonase (250 U/μl, EMD Millipore) for 1 hour. An aliquot (20 μl) of the lysate of each treatment group was saved as input. Lysates were resuspended in 800 μl of IP lysis buffer containing 2.5 μl of anti-FLAG M2 antibody (Sigma-Aldrich) and rotated overnight at 4°C. Fifty microliters of Protein A/G PLUS-agarose (Santa Cruz Biotechnology) slurry was added and incubated with the lysates for another 4 hours. After centrifugation, the immunoprecipitates were washed with RIPA buffer two times and then resuspended in 2 × Laemmli buffer for SDS-PAGE and immunoblotting with various antibodies as indicated.

### PAR Ab-IP-MS

The cells were washed 1 × PBS then lysed with alkaline lysis buffer (200 mM NaOH/2 and mM EDTA) on ice. The lysates were then neutralized by adding neutralizing buffer [1 M HCl/600 mM tris (pH 8.0)]. After neutralization, 10 × micrococcal nuclease reaction buffer (New England Biolabs), 100 U of micrococcal nuclease (New England Biolabs), and 100 × protease inhibitor cocktail (Thermo Fisher Scientific) were added to the samples (*59*, *60*), followed by PAR-IP and protein A/G agarose bead. The immunoprecipitates were processed for MS analysis.

### Molecular combing assay

Fork stability was analyzed using a molecular combing assay. Briefly, U2OS cells were labeled with CldU (100 μM) for 1 hour, and the cells were sequentially labeled with IdU (100 μM) together with treatment with FA (400 μM) and FEN1i (10 μM), and in combination for 1 or 4 hours. Next, the cells were trypsinized and mixed 1.5% low-melting agarose and then solidified as DNA plugs. The plugs were incubated in lysis buffer (Proteinase K (1 mg/ml), 100 mM EDTA, 1% sarkosyl, and 10 mM tris-Cl (pH 8.0)] overnight at 50°C. The next day, the plugs were gently washed with TE buffer and transferred into 1.6 ml of 0.1 M MES (pH 6.5) to melt agarose for 20 min at 70°C. The agarose was destructed by incubation with β-agarase (NEB) overnight at 42°C and transferred to slides to stretch DNA fibers. The slides were dried for 2 hours at 60°C and incubated with 0.4 M NaOH for 30 min. The slides were dehydrated and sequentially incubated with anti-mouse BrdU antibody (347580, BD Biosciences) and anti-rat BrdU antibody (ab6326, Abcam) overnight at 4°C. Following incubation with goat anti-rat secondary antibody (cy5; ab6565, Abcam) and goat anti-mouse secondary antibody (cy3; ab6946, Abcam) for 2 hours, the slides were incubated with anti-mouse single-stranded DNA primary antibody (MAB3034, Millipore) and secondary antibody BV480 (115-685-166, Jackson ImmunoResearch). The slides were mounted and scanned by FiberVison Automated Scanner (Genomic Vision). The relative ratio between CldU fibers and IdU fibers was calculated from captured images (NImage50/each) and plotted using GraphPad Prism version 9.2.0.

### Flow cytometry with EdU and DAPI

Replication activity was examined by using Click-iT Plus EdU Flow Cytometry Assay Kits according to the manufacturer’s instructions (C10634, Thermo Fisher Scientific). Briefly, the cells were labeled with EdU (10 μM) for 30 min before cell harvest. The cells then were fixed with 100 μl of Click-iT fixative (component D) and resuspended the cells in 100 μl of 1X Click-iT saponin-based permeabilization reagent. After the washing steps, fluorescent signals were confirmed by flow cytometry using a FACS Canto (Becton Dickinson). Data were analyzed using FlowJo Software.

### Neutral comet assay

The comet assays were performed according to the Trevigen CometAssay kit protocol with slight modifications. Cells were pretreated with PARGi for 1 hour, followed by cotreatment with 20 μM CPT for 2 hours. The treated cells were trypsinized at 37°C for 5 min. Equal amount of drug-free medium was then added to quench the trypsin activity. The cells were spun down and resuspended in fresh PBS. The final cell density was approximately 100,000 cells/ml. Fifty microliters of the cell suspension was then mixed with 500 μl of 0.5% low-melting point agarose (Invitrogen) (in PBS) at 37°C. Fifty microliters of the cell/agarose mixture was transferred onto glass slides. The slides were then immersed in prechilled lysis buffer [2.5 M NaCl, 100 mM EDTA, 10 mM tris (pH 10.0), 1% Triton X-100, and 10% Me_2_SO] for 1 hour. For alkaline comet assay, the slides were immersed in alkaline unwinding solution (200 mM NaOH and 1 mM EDTA) for 30 min at room temperature, followed by electrophoresis in 4°C alkaline electrophoresis solution (300 mM NaOH and 1 mM EDTA) at 1 V/cm for 30 min. For neutral comet assay, the slides were immersed in 1× Tris-borate-EDTA (TBE) buffer for electrophoresis at 1 V/cm for 30 min at room temperature. For both alkaline and neutral comet assays, the slides were immersed in 70% EtOH for 5 min after electrophoresis and then incubated with SYBR Gold for 30 min. The images were visualized under BioTek Cytation 5 cell imaging reader. Statistical analysis was performed by OpenComet, an ImageJ plugin.

### Instant structural illumination microscope

U2OS cells were transfected with FEN1-GFP and TOP1-HaloTag expression plasmids and exposed to FA (400 μM) for indicated time points following 48 hours of transfection. Live cells were imaged under a customized instant structural illumination microscope. For fluorescence imaging, 2 mM thymidine was added to U2OS cells in chamber slides at 37°C for 18 hours. Thymidine was removed, and fresh DMEM was added to the slides for incubation for 9 hours. Thymidine (2 mM) was added to the cells for another 18 hours of incubation. The cells are released from G_1_-S boundary by washing with PBS and incubating in fresh medium. PARGi (10 μM) and IdU (100 μM) were added to cells 1 hour before FA (400 μM) treatment. The cells were collected at indicated time points upon exposure to CPT and washed with PBS, followed by fixation with 4% PFA for 15 min at room temperature. The cells were then incubated with 1.5 M HCl for 30 min at room temperature for DNA denaturation, followed by permeabilization with 0.25% Triton X-100 in PBS (PBST). The cells were blocked with 1% BSA in 0.1% PBST for 30 min, followed by incubation with rabbit anti-γH2AX antibody and mouse anti-BrdU antibody overnight at 4°C. The next day, Alexa Fluor 568–conjugated anti-rabbit second antibody and Alexa Fluor 488–conjugated anti-mouse antibody were added to chamber slide for 1 hour at room temperature. The slides were incubated with DAPI and mounted using ProLong antifade mountant. Statistical analysis was performed by ImageJ software.

### Micronucleus analysis

MCF7 WT and shFEN1 cells seeded in chamber slide were exposed to either DMSO or 500 μM FA for 2 hours at 37°C. The cells were collected at indicated time points and washed with PBS, followed by fixation with 4% PFA for 15 min at room temperature. Chamber slides were mounted with DAPI, and interphase cells and micronuclei were measured with Nikon SoRa super-resolution spinning disk microscope.

### Purification of recombinant FEN1 proteins

FEN1 cDNA fragments carrying D181A mutation and E285Q mutation were inserted into a his-SUMO tag vector (Abclonal), respectively. The *E. coli* strain containing the correct plasmid was induced with 0.2% arabinose at 25°C overnight. After harvesting, the pellet was sonicated, and the supernatant was separated through centrifugation. The clean supernatant was incubated with HisPur Cobalt beads (Thermo Fisher Scientific). FEN1 proteins were eluted by 1 M imidazole buffer after washing the beads with a low concentration of imidazole buffer. Quality control was conducted by SDS-PAGE.

### Processing of PARylated protein samples for MS

Agarose beads containing immunoprecipitated proteins were centrifuged at 1500*g* for 1 min (room temperature), and supernatants were removed. The beads were suspended with 25 μl of S-Trap lysis buffer [5% SDS and 50 mM tetraethylammonium bromide (TEAB, pH 8.5)] and reduced with 20 mM tris (2-carboxyethyl) phosphine at 56°C for 1 hour. The samples were alkylated with 20 mM iodoacetamide at room temperature for 30 min in the dark. The samples were acidified with 2.5 μl of 27.5% phosphoric acid to completely denature the proteins. The samples were mixed with 170 μl of S-Trap binding buffer and loaded onto S-Trap columns (PROTIFI). The columns were centrifuged at 4000*g* for 30 s to allow the colloidal proteins to trap in the columns. The columns were washed four times with 200 μl of S-Trap binding buffer. The columns were again centrifuged at 4000*g* for 1 min to remove any residual buffers. Following washing, the trapped proteins in the columns were digested with 2 μg of trypsin suspended in 50 mM TEAB at 37°C for overnight. The next day, the tryptic peptides were sequentially eluted with 40 μl of 50 mM TEAB (pH 8.5), 0.2% formic acid, and 50% acetonitrile. The eluted samples were lyophilized and subjected to PAR modification removal as described below.

### Removal of PAR modification from tryptic peptides

The lyophilized peptides were suspended in PDE buffer [100 mM Hepes (pH 8.0) and 15 mM MgCl_2_]. Subsequently, 25 mg of peptides from each sample was treated with 4 mg of NudT16 enzyme at 37°C for 2 hours to remove PAR groups attached to the peptides. The treated peptides were finally desalted using C18 columns followed by liquid chromatography tandem MS.

### Colony formation assay

One day after transfection, the cells were diluted and seeded in six-well plates. One day after seeding, the cells were treated with indicated concentrations of ETOP or FA for 24 hours. The cells were then incubated for another 10 days before crystal violet staining and visualization. Plates were scanned by a ChemiDoc imaging system (Bio-Rad). Cell colonies were analyzed using ImageJ software.

### Statistical analyses

All experiments were repeated at least three independent times unless otherwise stated in the figure legend. Error bars on bar graphs represent SD except where stated otherwise. The *P* value was calculated using one-way analysis of variance (ANOVA) analysis followed by paired Student’s *t* test for independent samples. Bonferoni correction was used to make the overall type 1 error 0.05. Statistical analyses were performed using GraphPad Prism software.
