# Supplementary material for: Flap endonuclease 1 repairs DNA-protein cross-links via ADP-ribosylation–dependent mechanisms
Source: Sci Adv. 2025 Jan 10;11(2):eads2919. doi: 10.1126/sciadv.ads2919 (PMC11721697; doi:10.1126/sciadv.ads2919)
Supplement: Supplementary file 1 — Figs. S1 to S7 Legends for dataset S1 to S4 [file sciadv.ads2919_sm.pdf]

Science Advances
AAAS

Supplementary Materials for

Flap endonuclease 1 repairs DNA-protein cross-links via ADP-ribosylation–dependent mechanisms

Yilun Sun *et al.*

Corresponding author: Yilun Sun, yilun.sun@som.umaryland.edu

Sci. Adv. **11**, eads2919 (2025)
DOI: 10.1126/sciadv.ads2919

The PDF file includes:

Figs. S1 to S7
Legends for dataset S1 to S4

Other Supplementary Material for this manuscript includes the following:

Dataset S1 to S4

Supplementary figure 1

a

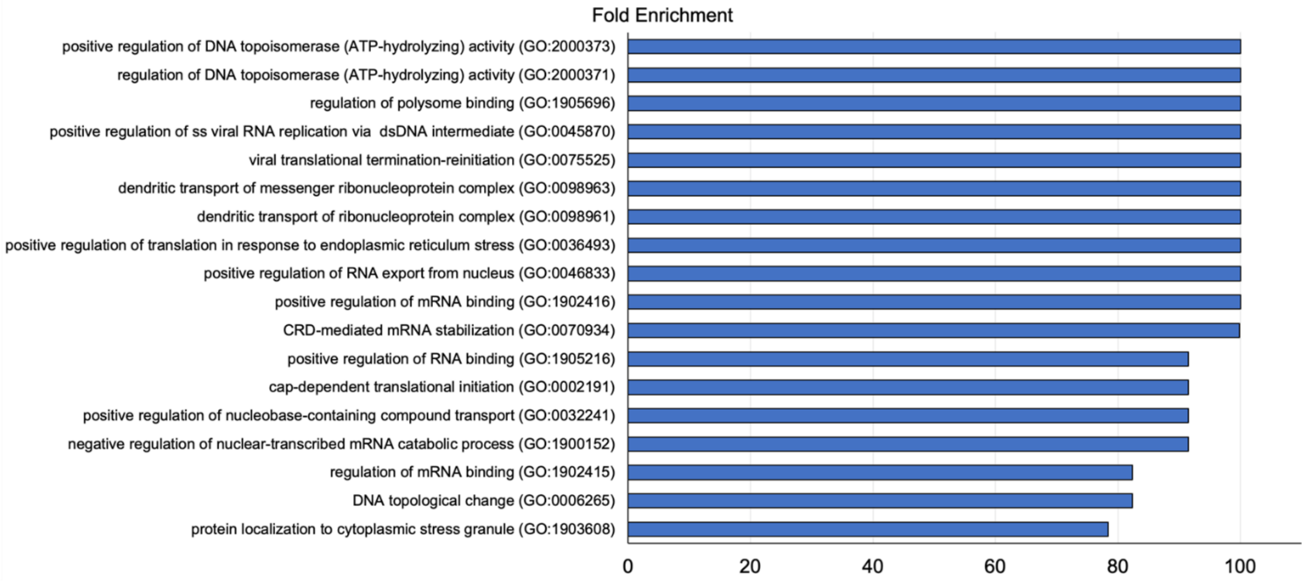

b

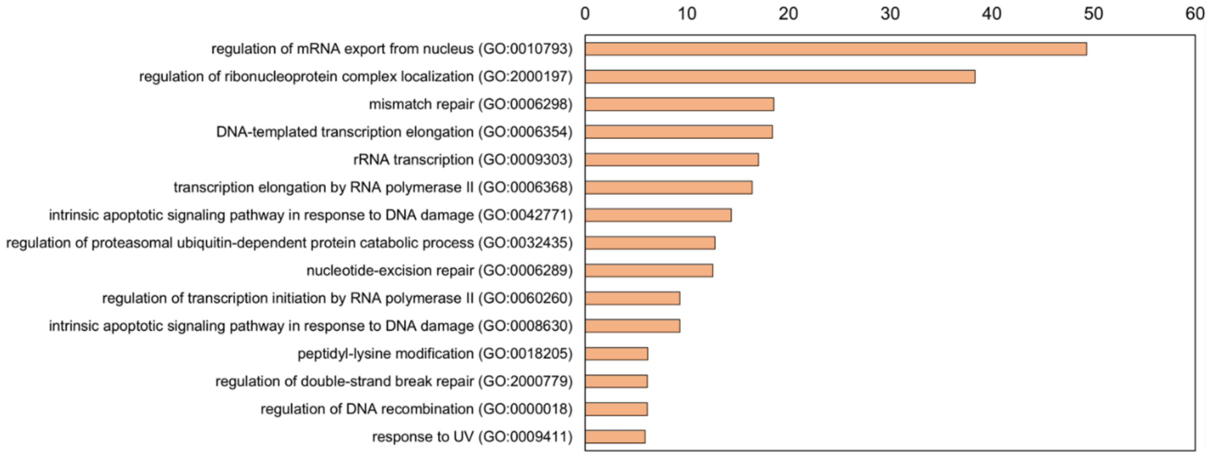

Supplementary Figure 1. The ICE-MS method identified the FA-induced DPC proteome.
a. The common FA-induced protein adducts across the three cell lines were mapped to the human proteome, which was annotated with the Gene Ontology (GO) biological process. Enrichment analysis was performed by fold change measurement. **b.** FA-enriched chromatin-bound proteins in the U2OS cell line were mapped to the human proteome, which was annotated with the Gene Ontology (GO) biological process. Enrichment analysis was performed by fold change measurement.

Supplementary figure 2

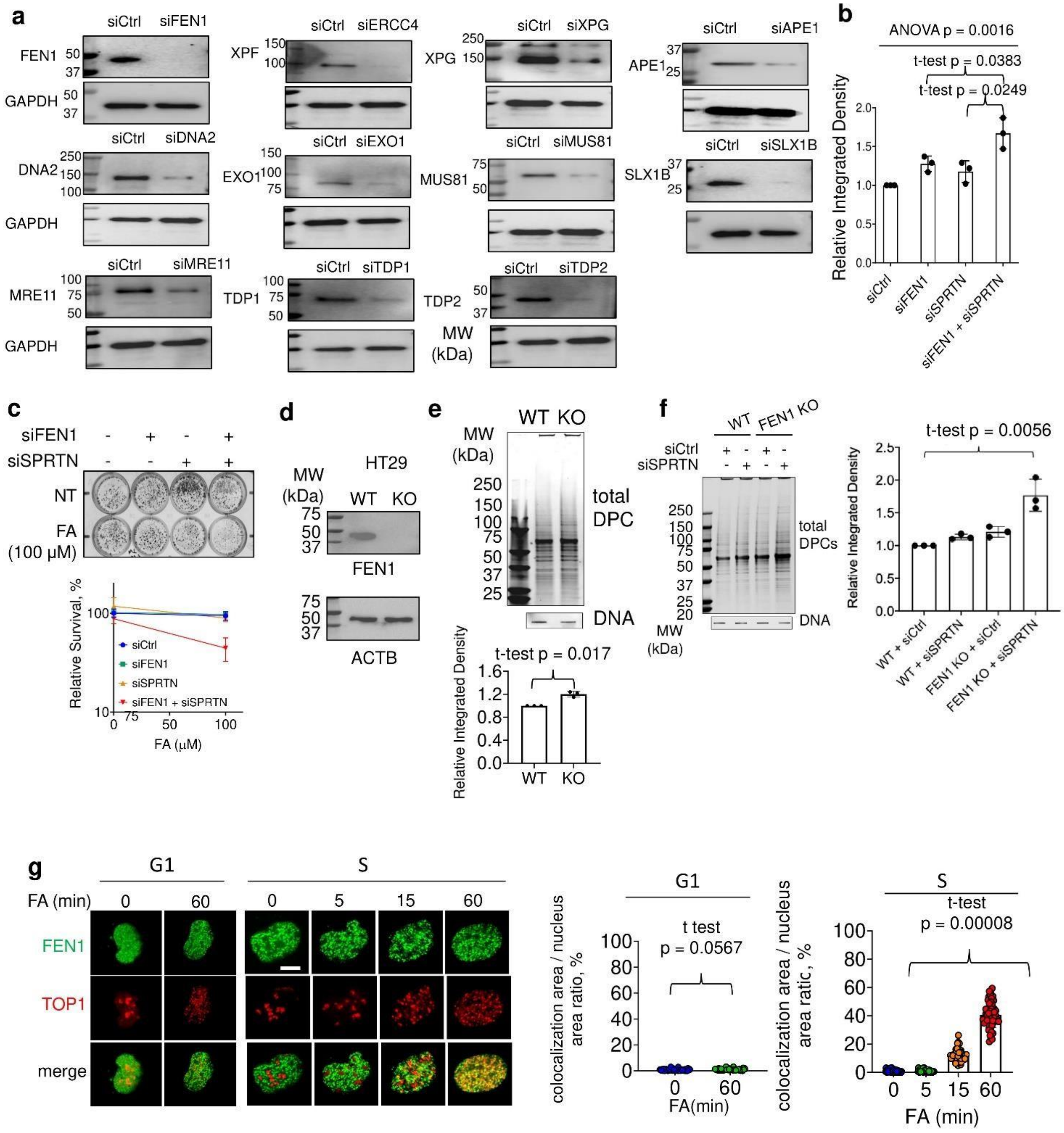

Supplementary Figure 2. FEN1 is a major nucleolytic mechanism for non-enzymatic DPC repair.

a. Western blotting in HEK293 cells confirming siRNA knockdown of genes tested by the modified RADAR assay in Fig. 2c by their respective antibodies. **b.** Densitometric analysis comparing total DPC signals generated from the modified RADAR assays including blot shown in Fig. 2c. Density of total DPCs/density of DNA of each group was normalized to cells treated with FA alone. n = 3 independent experiments. Data are presented as mean ± SD. **c.** Colony formation assay was conducted in MCF7 cells transfected with indicated siRNAs. Following 48 h transfection, cells were exposed to 20 μM FA for 24 h and replenished with drug-free media. Colony numbers were measured using ImageJ. **d.** Western blotting in HT29 cells confirming the CRISPR knocking-out of FEN1. **e. Upper panel:** The modified RADAR assay was performed in WT and FEN1 KO HT29 colon cell line transfected with indicated siRNAs for 48 hrs. 20 μg samples were digested with 100 units of micrococcal nuclease and subjected to SDS-PAGE electrophoresis. Total DPCs were detected with Coomassie stain. 2 μg samples without micrococcal nuclease digestion were subjected to slot-blot and probed with anti-DNA antibody as loading control. **Lower panel:** Densitometric analysis comparing total DPC signals generated from the modified RADAR assays including the blot shown in the upper panel. The density of total DPCs/density of DNA of each group was normalized to HT29 WT cells. n = 3 independent experiments. Data are presented as mean ± SD. **f.** Left panel: The modified RADAR assay was performed in WT and FEN1 KO HT29 colon cell line transfected with indicated siRNAs for 48 hrs. 20 μg samples were digested with 100 units micrococcal nuclease and subjected to SDS-PAGE electrophoresis. Total DPCs were detected with Coomassie stain. 2 μg samples without micrococcal nuclease digestion were subjected to slot-blot and probed with anti-DNA antibody as loading control. Right panel: Densitometric analysis comparing total DPC signals generated from the modified RADAR assays including blot shown in the left panel. Density of total DPCs/density of DNA of each group was normalized to HT29 WT cells transfected with siCtrl. n = 3 independent experiments. Data are presented as mean ± SD. **g. Left panel:** Instant structure illumination microscopy analysis in live U2OS cells (arrested in the G1 or S phase) transfected with TOP1-HaloTag and FEN1-GFP constructs and subsequently exposed to 400 μM FA for indicated periods. The scale bar represents 10 μm. **Right panel:** Colocalization area and nucleus area in each cell including the representative cells in the left panel were measured by ImageJ color threshold and the level of colocalization in each cell was determined. n = 200 biologically independent cells.

Supplementary figure 3

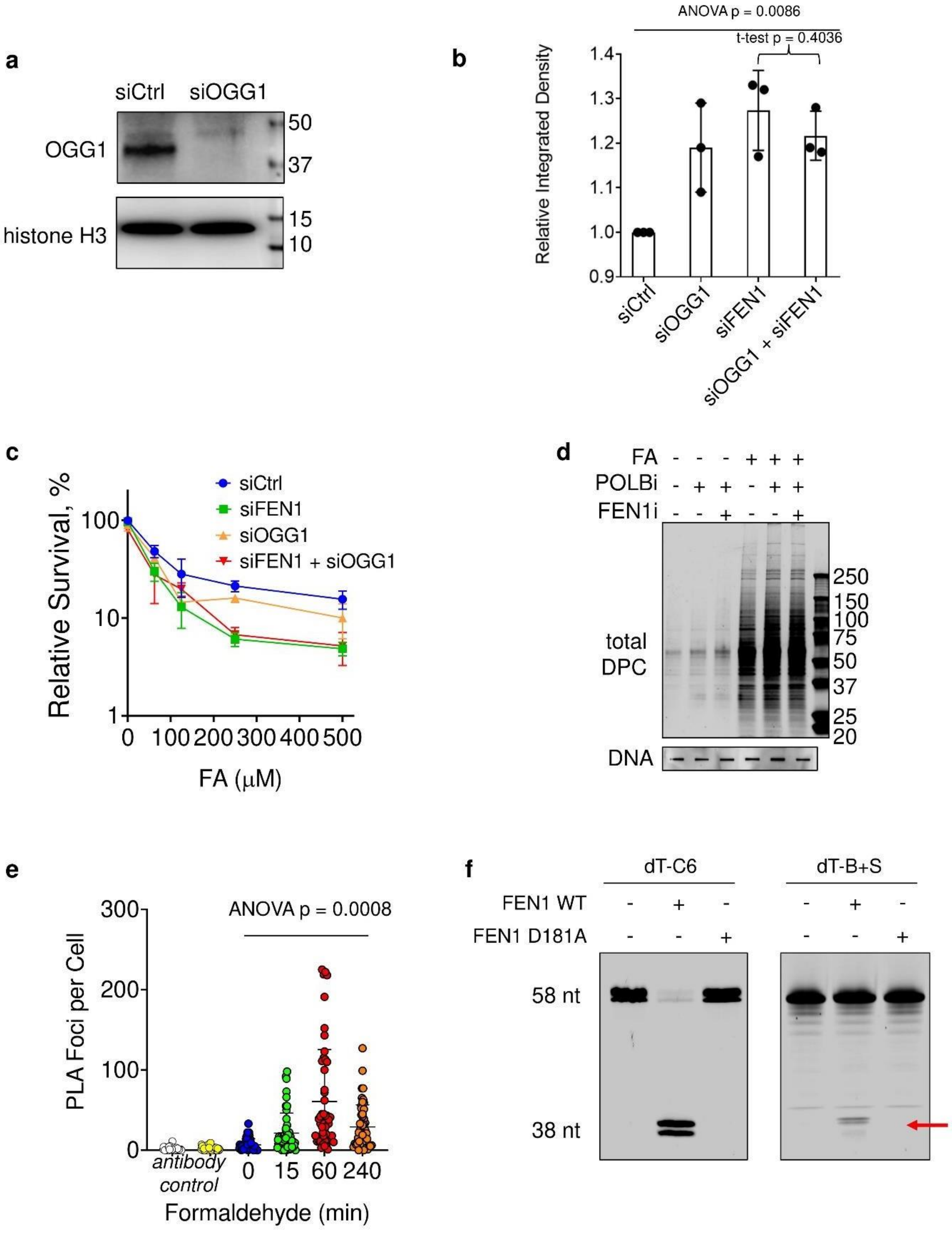

Supplementary Figure 3. FEN1 cleaves DPC-harboring DNA flaps originated oxidative lesions.

a. Western blotting in HEK293 cells confirming siRNA knockdown of OGG. **b.** Densitometric analysis comparing total DPC signals generated from the modified RADAR assays in Fig. 3d. Density of total DPCs/density of DNA of each group was normalized to cells transfected with control siRNA (siCtrl). n = 3 independent experiments. Data are presented as mean ± SD. **c.** Viability curve derived from ATPlite luminescence assay in MCF7 cells transfected with indicated siRNAs and treated with FA at indicated concentrations for 72 h (mean ± SD, n = 3). **d.** The modified RADAR assay was performed in U2OS cells treated with indicated drugs. Total DPCs were detected with Coomassie stain. **e.** Quantitation of PLA foci indicating TOP1 and 8-OXO-dG interaction with mean ± SD using Thunderstorm, a plugin of ImageJ. Data were obtained from experiments shown in Fig. 3E. n = 200 biologically independent cells. **f.** Activity assay testing indicated recombinant human FEN1 proteins towards the C6-modified DNA substrate (left panel) and streptavidin-biotin-modified DNA substrate (right panel) for 30 min. Cy5 labeled DNA products following the activity assay were visualized by PAGE electrophoresis.

Supplementary figure 4

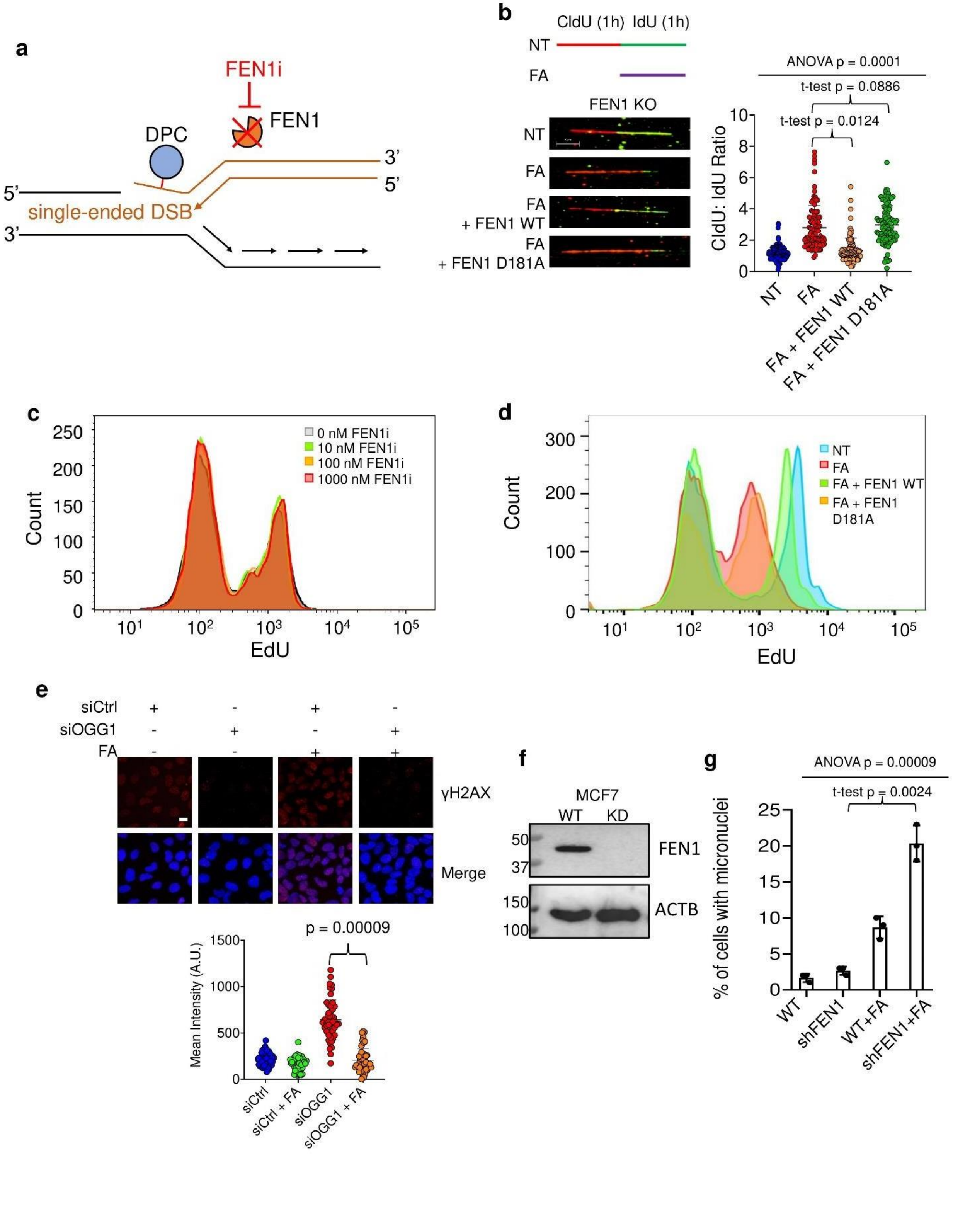

Supplementary Figure 4. FEN1 prevents FA-induced replication stress and chromosomal instability.

a. Hypothetical model depicting the induction of DSBs by FA. Ongoing replication forks, once colliding with 5'-flap DPCs ahead of the forks in the absence of FEN1 activity, can be converted into single-ended DSBs that stall replication. **b. Left upper panel:** labeling protocols for DNA combing assay in HT29 FEN1 CRISPR KO cells. FA: 400 µM. **Left lower panel:** Representative images of CldU and IdU tracks from combing assays performed under conditions described in the upper panel. **Right panel:** CIdU/IdU Ratio with mean ± SD measured from experiments shown in the left panel. **c.** EdU incorporation was analyzed by flow cytometry. Cells were treated with FEN1i at indicated concentrations for 4 h and pulsed with EdU (10 µM) for 30 min prior to harvesting. **d.** EdU incorporation was analyzed by flow cytometry. Cells were treated with 400 µM FA for 1 h and pulsed with EdU (10 µM) for 30 min before harvesting. **e. Upper panel:** representative images of IF of γH2AX foci by confocal microscopy. U2OS cells transfected with control siRNA or OGG1 siRNA for 48 hours were synchronized in S-phase by double thymidine block, followed by 400 µM FA treatment for 4 hours for IF using anti-γH2AX antibody. The scale bar represents 20 µm. **Lower panel:** Quantitation of γH2AX intensity from experiments shown in the upper panel using ImageJ. n = 200 biologically independent cells. **f.** Western blotting in MCF7 cells confirms the knockdown of FEN1. **g.** Quantification of micronucleus containing interphase cells (error bars = SD) from experiments shown in Fig. 4e.

Supplementary figure 5

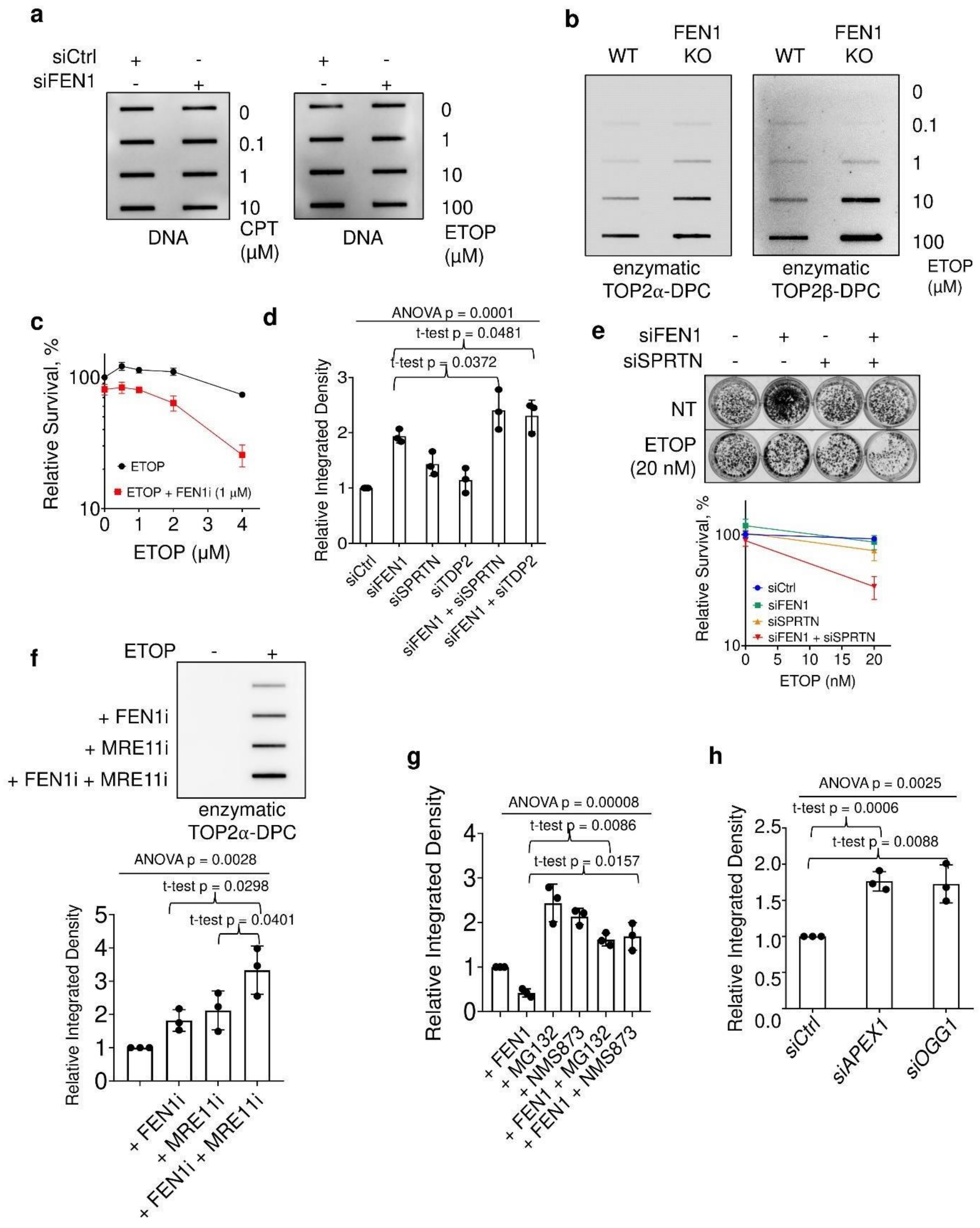

Supplementary Figure 5. FEN1 repairs enzymatic TOP2-DPCs independently of the BER pathway.

a. DNA slot-blot measured by the ICE assay in Fig. 5a. **b.** The RADAR assay was performed in HT29 WT and FEN1 KO cells treated with ETOP of indicated concentrations for 1 h for immunodetection with indicated antibodies. **c.** Viability curve derived from ATPlite luminescence assay in MCF7 cells treated ETOP with or without 1 μM FEN1 inhibitor FEN1-IN-4 at indicated concentrations for 72 h (mean ± SD, n = 3). **d.** Densitometric analysis comparing TOP2α-DPC signals generated from the RADAR assays including blot shown in Fig. 5D. Density of TOP2α-DPCs /density of DNA of each group were normalized to cells transfected with control siRNA (siCtrl). n = 3 independent experiments. Data are presented as mean ± SD. **e.** MCF7 cells were transfected with indicated siRNAs for 48 h before ETOP treatment (200 nM, 24 h). The relative cellular sensitivity to ETOP treatment of indicated cells was determined by colony formation assay. **f. Upper panel**: The RADAR assay was performed in HEK293 cells pre-treated with indicated inhibitors (FEN1i: FEN1-IN-4, 1 μM, 2h; MRE11i: PFM01, 1 μM, 2h) and then co-treated with 10 μM ETOP for 1 h. **Down panel:** Densitometric analysis comparing TOP2α-DPC signals generated from the RADAR assays including blot shown in the left panel. The density of TOP2α- DPCs /density of DNA of each group was normalized to cells treated with ETOP alone. n = 3 independent experiments. Data are presented as mean ± SD. **g.** Densitometric analysis comparing TOP2α-DPC signals generated from the RADAR assays including blot shown in Fig. 5e. The density of TOP2α-DPCs /density of DNA of each group was normalized to cells treated with ETOP alone. n = 3 independent experiments. Data are presented as mean ± SD. **h.** Densitometric analysis comparing TOP2α- DPC signals generated from the RADAR assays including blot shown in Fig. 5f. The density of TOP2α-DPCs /density of DNA of each group were normalized to cells transfected with control siRNA (siCtrl). n = 3 independent experiments. Data are presented as mean ± SD.

Supplementary figure 6

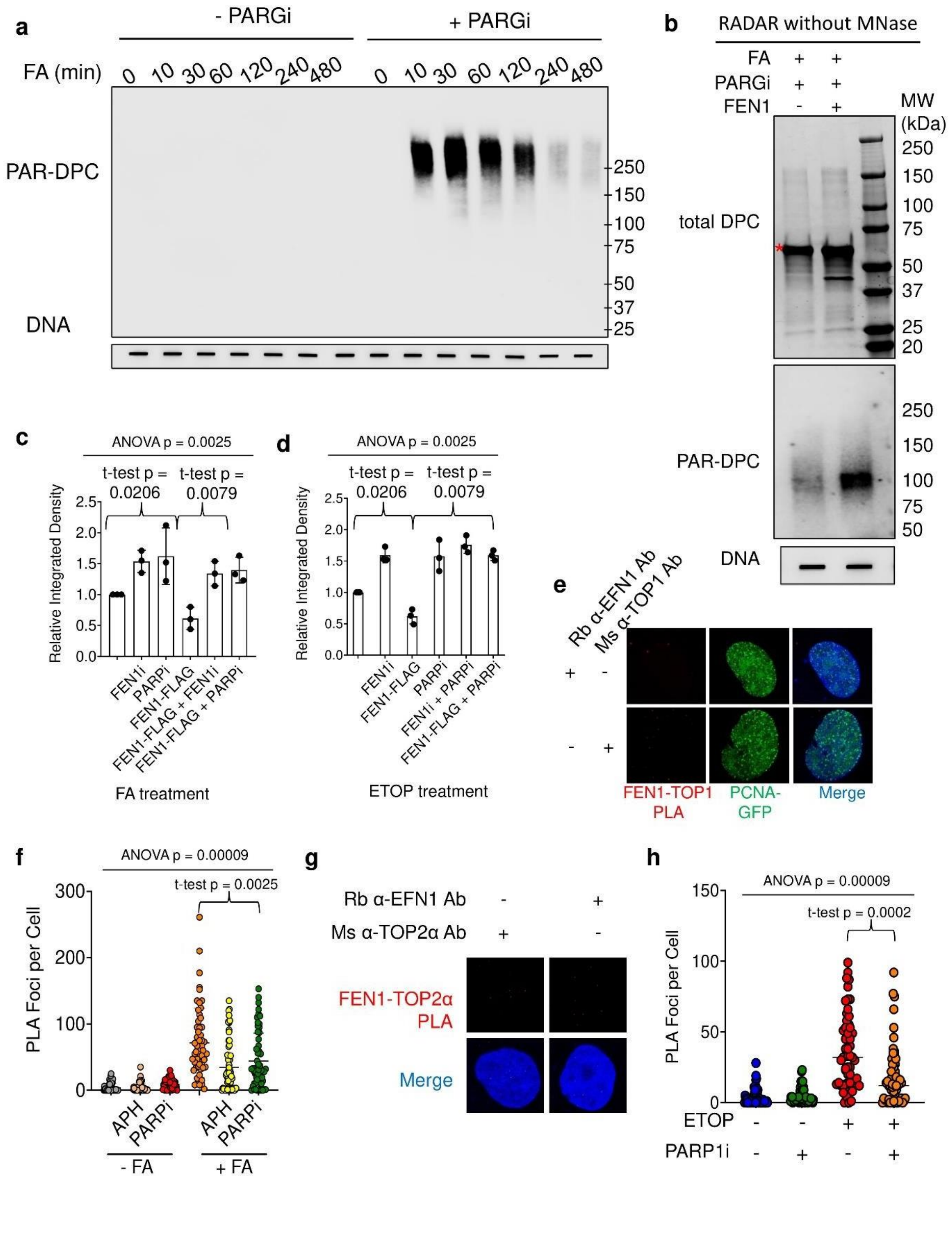

Supplementary Figure 6. PARP1 induces FEN1-dependent repair of non-enzymatic and enzymatic DPCs.

a. HEK293 cells pre-treated with 10 μM PARGi for 1 h then co-treated with 400 μM FA for indicated periods, followed by the modified RADAR assay to detect the kinetics of DPC PARylation using an anti-PAR antibody. **b.** HEK293 cells were pre-treated with 10 μM PARGi for 1h and then co-treated with 400 μM FA for 2 h, followed by the modified RADAR assay. Instead of micrococcal nuclease, the RADAR samples were digested with or without recombinant FEN1 in FEN1 cleavage buffer before SDS-PAGE electrophoresis. Total DPCs were detected by Coomassie stain and PARylated DPCs were probed with anti-PAR antibody. *, BSA. **c.** Densitometric analysis comparing total DPC signals generated from the modified RADAR assays including blot shown in Fig. 6C. Density of total DPCs/density of DNA of each group was normalized to cells treated with FA alone. n = 3 independent experiments. Data are presented as mean ± SD. **d.** Densitometric analysis comparing TOP2α-DPC signals generated from the RADAR assays including blot shown in Fig. 6D. Density of TOP2α-DPCs/density of DNA of each group was normalized to cells treated with ETOP alone. n = 3 independent experiments. Data are presented as mean ± SD. **e.** Assay controls for Fig. 6e. **f.** Quantitation of PLA foci indicating TOP1-FEN1 interaction with mean ± SD using Thunderstorm. Data were obtained from experiments shown in Fig. 6e. n = 200 biologically independent cells. **g.** Assay controls for Fig. 6e. **h.** Quantitation of PLA foci indicating TOP2α-FEN1 interaction with mean ± SD using Thunderstorm. Data were obtained from experiments shown in Fig. 6f. n = 200 biologically independent cells.

Supplementary figure 7

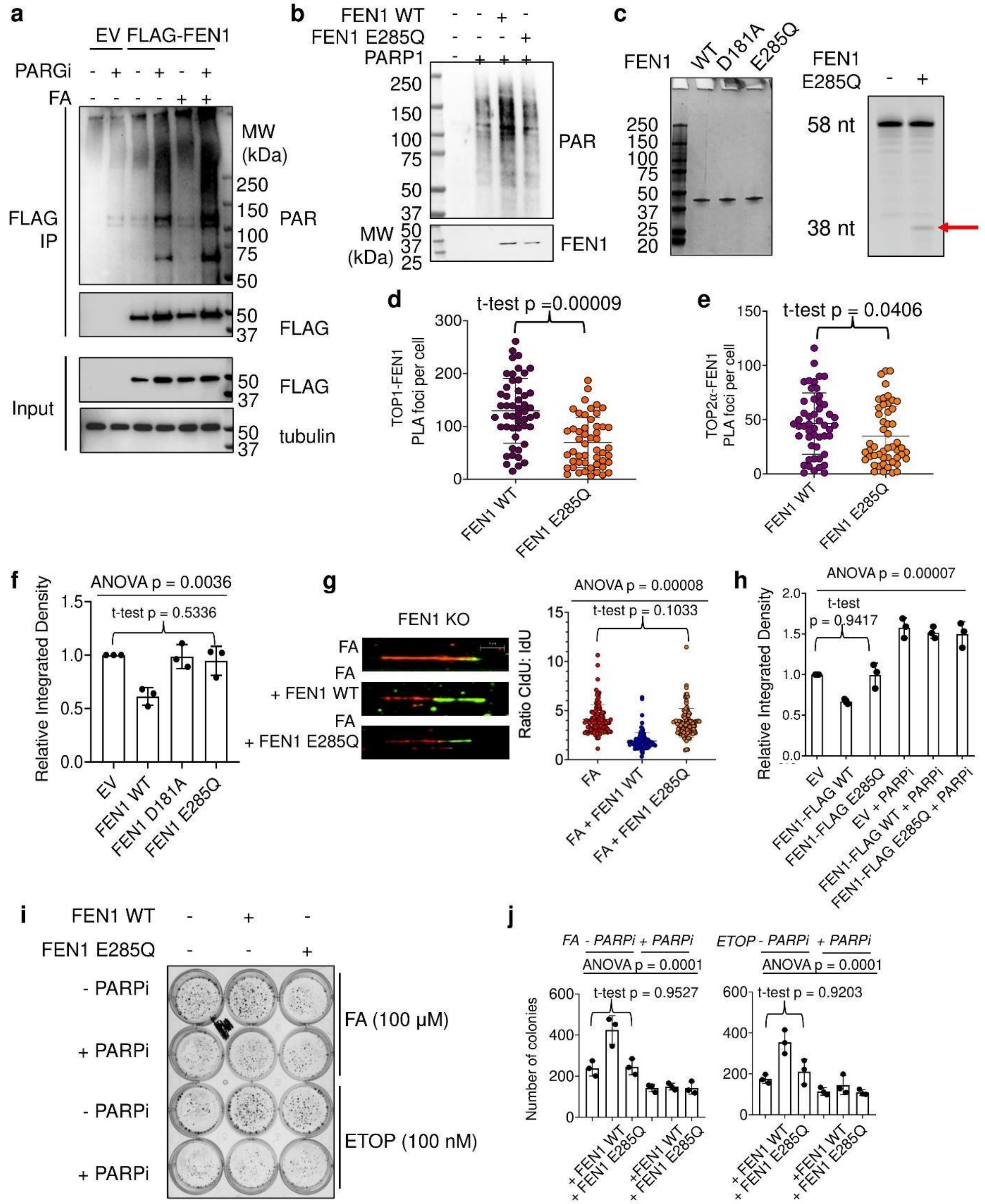

Supplementary Figure 7. ADP-ribosylation of FEN1 at glutamic acid 285 residue localizes FEN1 to DPC sites for repair.

a. Following transfection of empty vector (EV) or FEN1-FLAG expression plasmid, HEK293 cells were treated with the indicated drugs (10 µM PARGi pre-treatment for 1 h followed by 1 h co-treatment with 400 µM FA) and subjected to IP using anti-FLAG antibody. The immunoprecipitates and input samples were Western blotted with the indicated antibodies. **b.** *in vitro* PARylation assay with recombinant WT FEN1 protein or FEN1 E285Q protein, recombinant PARP1 protein, NAD^+, and activated DNA. Following 20 min incubation at room temperature, samples were subjected to Western blotting using anti-PAR and anti-FEN1 antibodies. **c. Left panel**: Coomassie blue stining of recombinant FEN1 WT and mutants. **Right panel:** Activity assay testing indicated recombinant human FEN1 E285Q mutant protein towards streptavidin-biotin- modified DNA substrate for 30 min. Cy5 labeled DNA products following the activity assay were visualized by PAGE electrophoresis. **d.** Quantitation of PLA foci indicating TOP1-FEN1 interaction with mean ± SD using Thunderstorm. Data were obtained from experiments shown in Fig. 7e. n = 200 biologically independent cells. **e.** Quantitation of PLA foci indicating TOP2α- FEN1 interaction with mean ± SD using Thunderstorm. Data were obtained from experiments shown in Fig. 7f. n = 200 biologically independent cells. **f.** Densitometric analysis comparing total DPC signals generated from the modified RADAR assays including blot shown in Fig. 7g. Density of total DPCs/density of DNA of each group was normalized to cells treated with FA but without FEN1-FLAG expression plasmid transfection (empty vector, EV). n = 3 independent experiments. Data are presented as mean ± SD. **g.** DNA combing assay in HT29 FEN1 CRISPR KO cells treated with 400 µM FA. The labeling protocol is the same as the one used in Supplementary Fig. 4b. Left lower panel: Representative images of CldU and IdU tracks from combing assays. Right panel: CIdU/IdU Ratio with mean ± SD measured from experiments shown in the left panel. **h.** Densitometric analysis comparing TOP2α-DPCs generated from the RADAR assays including blot shown in Fig. 7h. Density of TOP2α-DPC/density of DNA of each group was normalized to cells treated with ETOP but without FEN1-FLAG expression plasmid transfection (empty vector, EV). n = 3 independent experiments. Data are presented as mean ± SD. **i.** HT29 FEN1 CRISPR KO cells were transfected with indicated plasmids for 48 h before FA (20 µM, 24 h) or ETOP treatment (200 nM, 24 h) with or without co-treatment with PARPi olaparib (10 nM). **j.** The relative cellular sensitivity was determined by colony formation assay. Colony numbers were measured using Image J.

Supplementary dataset 1. Excel files of the proteome of FA-induced DNA-proteins crosslinks in three human cell lines by the ICE-MS.

Supplementary dataset 2. Excel files of the proteome of chromatin fraction with or without FA treatment in human osteosarcoma U2OS cells.

Supplementary dataset 3. Excel files of the z-score analysis for the RNAi screening in human breast cancer MCF7 cells.

Supplementary dataset 4. Excel files of the proteome of PARylated proteins with or without FA treatment in human embryonic kidney HEK293 cells.
